# Fermi surface in La-based cuprate superconductors from Compton scattering imaging

**DOI:** 10.1038/s41467-021-22229-6

**Published:** 2021-04-13

**Authors:** Hiroyuki Yamase, Yoshiharu Sakurai, Masaki Fujita, Shuichi Wakimoto, Kazuyoshi Yamada

**Affiliations:** 1 International Center for Materials Nanoarchitectonics, National Institute for Materials Science (NIMS), Tsukuba, Japan; 2 Department of Condensed Matter Physics, Graduate School of Science, Hokkaido University, Sapporo, Japan; 3 Japan Synchrotron Radiation Research Institute (JASRI), Hyogo, Japan; 4 Institute for Materials Research, Tohoku University, Sendai, Japan; 5 Materials Sciences Research Center, Japan Atomic Energy Agency, Tokai, Naka, Ibaraki Japan; 6 High Energy Accelerator Research Organization (KEK), Tsukuba, Japan

**Keywords:** Electronic properties and materials, Superconducting properties and materials

## Abstract

Compton scattering provides invaluable information on the underlying Fermi surface (FS) and is a powerful tool complementary to angle-resolved photoemission spectroscopy and quantum oscillation measurements. Here we perform high-resolution Compton scattering measurements for La_2−*x*_Sr_*x*_CuO_4_ with *x* = 0.08 (*T*_*c*_ = 20 K) at 300 K and 150 K, and image the momentum distribution function in the two-dimensional Brillouin zone. We find that the observed images cannot be reconciled with the conventional hole-like FS believed so far. Instead, our data imply that the FS is strongly deformed by the underlying nematicity in each CuO_2_ plane, but the bulk FSs recover the fourfold symmetry. We also find an unusually strong temperature dependence of the momentum distribution function, which may originate from the pseudogap formation in the presence of the reconstructed FSs due to the underlying nematicity. Additional measurements for *x* = 0.15 and 0.30 at 300 K suggest similar FS deformation with weaker nematicity, which nearly vanishes at *x* = 0.30.

## Abstract (web summary)

Compton scattering provides information on the Fermi surface (FS) hence very useful to understand the electronic structure of high temperature superconductors. Here, Yamase et al. perform Compton scattering measurements on La_2−x_Sr_*x*_CuO_4_ samples and observe deformed FS in CuO_2_ plane due to nematicity but recovering fourfold symmetry in bulk FS.

## introduction

The Fermi surface (FS) is a direct consequence of the Pauli exclusion principle and is very fundamental in the condensed matter physics. In particular, the shape of the FS reflects the electron motion inside material and is a key to control the low-energy properties of metals.

High-temperature cuprate superconductors are interesting metals and exhibit various phenomena by changing carrier doping and temperature^1^: the pseudogap phase, nematic order, charge-density-wave, spin-density-wave, and superconductivity. Although no consensus has been obtained on the coherent understanding of those phenomena, it is well recognized that the FS has a major role to understand the complicated physics in cuprates. However, the underlying FS in high-temperature cuprate superconductors remains elusive and controversial.

By applying a high magnetic field and suppressing the superconductivity, quantum oscillation measurements revealed that the large FS in the overdoped region is reconstructed to small Fermi pockets in the underdoped region at low temperature^2^. This reconstruction was interpreted to be driven by translation symmetry breaking owing to a charge-density-wave instability in the magnetic field. Recent Hall number measurements^3^ suggested that the charge-density-wave scenario alone cannot be a whole story. The FS reconstruction can be a consequence of both spiral (not collinear^4^) magnetic order and charge-density-wave in the high field^5^. Angle-resolved photoemission spectroscopy (ARPES) can reveal the FS in a condition without a magnetic field^6^. A clear signature of the reconstruction of the FS is not obtained so far. Rather a large FS seems to be present at least at high temperature and the spectral weight around the anti-nodal regions is substantially suppressed with decreasing temperature, leading to Fermi arcs around the nodal region at low temperature^7^. The origin of the Fermi arcs is directly related to the enigmatic phenomenon of the pseudogap, one of the most mysterious problems in high-*T*_*c*_ cuprate superconductors^8^. The consistent understanding of quantum oscillation data and ARPES data are not obtained and the underlying FS in cuprates remains to be studied.

ARPES measures the one-particle spectral function *A*(**k**, *ω*) and the underlying FS is usually obtained by tracing a peak position of *A*(**k**, *ω*) at *ω* = 0. However, *A*(**k**, *ω*) features a broad structure especially near the anti-nodal region owing to the pseudogap phenomenon^9^. A technique complementary to ARPES is Compton scattering. It measures the momentum distribution function *n*(**k**), which is obtained by integrating the product of *A*(**k**, *ω*) and the Fermi distribution function with respect to energy *ω*. This feature can in turn become an advantage over ARPES, because *n*(**k**) can be affected less severely by the opening of the gap around the anti-nodal region. In fact, the underlying FS can be inferred from *n*(**k**) even in the presence of a gap owing to superconductivity^10^ and spin-density-wave^11^. Furthermore, in contrast to ARPES, no matrix-element effect occurs and *n*(**k**) is imaged directly by Compton scattering in the whole Brillouin zone. As a result, we may reveal the underlying FS including the anti-nodal region by employing the Compton scattering technique. Compton scattering is actually a powerful probe to study fermiology. It is neither surface sensitive, in contrast to ARPES, nor disorder nor temperature-sensitive unlike quantum oscillation measurements. Compton scattering successfully revealed the FS in La_2−*x*_Sr_*x*_CuO_4_ (LSCO) with *x* = 0.3 (ref. ^12^), Sr_2_RuO_4_ (ref. ^13^), CeRu_2_Si_2_ (ref. ^14^), lithium^15^, palladium-hydrogen^16^, and various disordered alloys such as Li-Mg (ref. ^17^) and Cu-Pd (ref. ^18^).

In this paper, we report high-resolution x-ray Compton scattering for LSCO with *x* = 0.08. We find that the obtained momentum distribution function cannot be interpreted in terms of the conventional hole-like FS believed so far for La-based cuprates. A natural understanding is obtained by invoking the underlying electronic nematic correlations and their coupling to a soft phonon mode, which leads to a strongly deformed FS with *d*-wave symmetry in each CuO_2_ plane and its alternate stacking along the *z* axis. Additional Compton scattering measurements for *x* = 0.15 and 0.30 suggest similar FS deformation with weaker nematicity, which nearly vanishes at *x* = 0.30.

Our choice of *x* = 0.08 is made judiciously. As is well known, LSCO exhibits both spin^19^ and charge^20^ orders in 0.10 ≲ *x* ≲ 0.13. This state is discussed in terms of spin-charge stripe order^21^, where incommensurate magnetic order with wave vectors **q**_*s*_ = (*π*, *π* ± 2*πη*) and (*π* ± 2*πη*, *π*) coexists with one-dimensional charge stripe order with **q**_*c*_ ≈ ± 2**q**_*s*_; we use the tetragonal notation in this paper. Moreover, the spin glass phase, diagonal spin-stripe phase, and antiferromagnetic phase are realized in a low temperature and low doping region^22,23^. Given that the present measurement is the first Compton scattering study to explore the underlying FS in the underdoped LSCO, apparent complications should be safely avoided. We, therefore, choose *x* = 0.08 and a relatively high-temperature region.

## Results

**Fig. 1:**
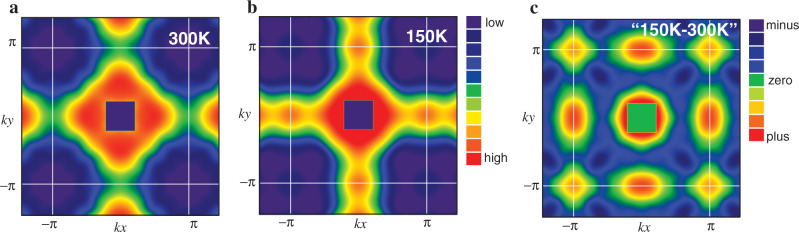
Images of the momentum distribution function *n*(**k**) by high-resolution Compton scattering. Maps of *n*(**k**) in the first Brillouin zone at 300 K (**a**) and 150 K (**b**) for La_2−*x*_Sr_*x*_CuO_4_ with *x* = 0.08. The color scale represents the relative intensity. **c** The difference of *n*(**k**) between 300 K and 150 K.

We perform high-resolution Compton scattering for LSCO with *x* = 0.08 at 300 K and 150 K. The momentum distribution function is then imaged in the two-dimensional Brillouin zone in Fig. 1a, b by applying the Lock-Crisp-West (LCW) theorem^24^: higher intensity indicates a region where electrons are occupied more in momentum space. Although all electrons contribute to the momentum distribution function in Compton scattering measurements, the momentum dependence of *n*(**k**) originates from the band crossing the Fermi energy. Therefore, *n*(**k**) shown in Fig. 1a, b reflects the underlying FS. The square region around **k** = (0, 0) contains a large experimental error and thus is not considered; see Methods section for details.

Usually, the spectrum of *n*(**k**) exhibits a weak temperature dependence even if a phase transition such as superconductivity occurs as long as translational symmetry is kept. However, as shown in Fig. 1a, b, the present work reveals for the first time a strong temperature dependence of *n*(**k**) for high-*T*_*c*_ cuprate superconductors. Figure 1c is the difference of *n*(**k**) between 300 K and 150 K, showing that *n*(**k**) is enhanced around **k** = (*π*, 0) and (0, *π*) at 150 K. As the pseudogap formation occurs below 200 K (ref. ^25^), this change should be related to the pseudogap. However, the pseudogap is pronounced around **k** = (*π*, 0) and (0, *π*), which would then suppress *n*(**k**) there as long as the FS is hole-like, in contrast to the observation in Fig. 1c. This apparent puzzle is solved by considering a combined effect of large FS deformation from the underlying nematicity and a broadening of *n*(**k**) owing to the pseudogap formation as we will analyze the data below. The enhancement around **k** = (*π*, *π*) in Fig. 1c can be a consequence of the charge conservation.

For cuprates, it is an open question how *n*(**k**) should depend on **k** both theoretically and experimentally; see Supplementary Note 1 for a general feature of *n*(**k**). Let us suppose a FS proposed by ARPES or tight-binding fitting and superpose it on our data of *n*(**k**). In this case, one naturally expects that *n*(**k**) > *n*(**k**_*F*_) [*n*(**k**) < *n*(**k**_*F*_)] for **k** inside (outside) the FS and *n*(**k**) ~ 0.5 at **k** = **k**_*F*_; here **k**_*F*_ is the Fermi momentum. Therefore, we reasonably assume that *n*(**k**_*F*_) should not exhibit a strong **k**_*F*_ dependence. However, this criterion itself may not be enough to discuss the underlying FS because the value of *n*(**k**_*F*_) cannot be exactly a certain value around 0.5. To compensate for this uncertainty, we also study a peak position of the absolute value of the first derivative of *n*(**k**), which should trace the underlying FS (ref. ^13^). In addition, we assume Luttinger’s theorem that the volume enclosed by the FS is equal to the electron density. Note that our proposed FSs in the nematic scenario (see below) also fulfill Luttinger’s theorem.

### Conventional scenario

**Fig. 2:**
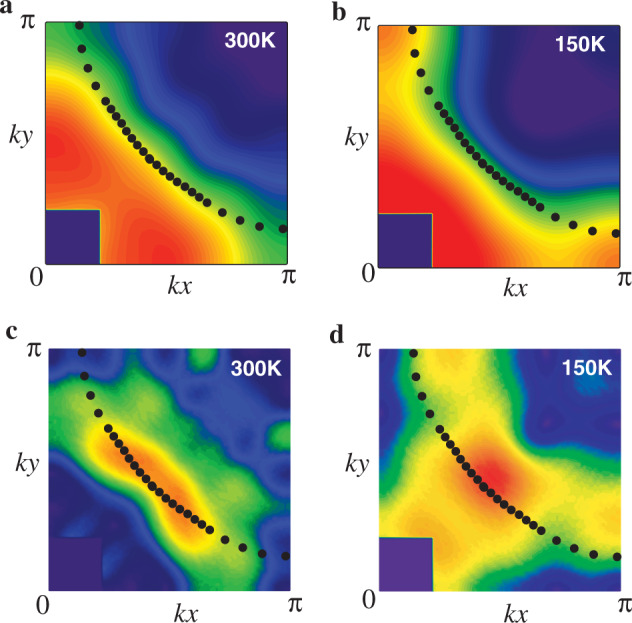
Interpretation of Fig. 1a, b in terms of the conventional Fermi surface (FS) (dots) reported by angle-resolved photoemission spectroscopy (ARPES) (ref. ^9^). **a** and **c** are the momentum distribution function *n*(**k**) and the magnitude of its derivative ∣ ∇ *n*(**k**)∣ at 300 K in the first quadrant of the Brillouin zone, respectively; **b** and **d** are the corresponding results at 150 K. Although the ARPES measurements were performed for La_2−*x*_Sr_*x*_CuO_4_ with *x* = 0.07 (ref. ^9^), the doping difference by 1% from the present study provides no visible change of the FS on the scale of the figure.

We first consider the conventional hole-like FS, which is believed to be realized in La-based cuprates^9^. As shown in Fig. 2a, b, a comparison between our Compton data and the conventional FS reveals that *n*(**k**_*F*_) exhibits a very weak **k**_*F*_ dependence in an extended region around **k** = (0.45*π*, 0.45*π*), consistent with the ARPES data^9^. However, a sizable **k**_*F*_ dependence is recognized in **k** ≈ (0.2*π*, 0.7*π*) − (0.1*π*, *π*) and its equivalent regions at both 300 K and 150 K. This is not consistent with a general understanding that *n*(**k**_*F*_) should not exhibit a strong **k**_*F*_ dependence.

Figure 2c, d shows maps of the magnitude of the first derivative of *n*(**k**), namely ∣ ∇ *n*(**k**)∣ at 300 K and 150 K, respectively, and the conventional FS is superposed there. At 300 K, the peak of ∣ ∇ *n*(**k**)∣ forms around **k** = (0.45*π*, 0.45*π*) with an inward curvature, suggesting the presence of the electron-like FS around **k** = (0, 0). However, the conventional FS has the opposite curvature, not consistent with our data. At 150 K, the discrepancy between our data and the conventional FS is reduced. But an agreement is still not so satisfactory because the map of ∣ ∇ *n*(**k**)∣ has a peak around (0.3*π*, *π*) and (*π*, 0.3*π*), whose position is largely away from the conventional FS. We should not consider the weak peak structure around (0.2*π*, 0.2*π*) in Fig. 2d, which can be an artifact coming from a large experimental error around **k** = (0, 0).

Considering both *n*(**k**) (Fig. 2a, b) and ∣ ∇ *n*(**k**)∣ (Fig. 2c, d), therefore, it is not possible to reconcile with the conventional FS both at 300 K and 150 K. To explore a possible clue to resolve such a problem, we first checked that the temperature dependence of the chemical potential is indeed negligible; see also Supplementary Note 3. Although one might then wonder about the effect of the thermal broadening of *n*(**k**), it is also not relevant to the present analysis; see Supplementary Note 4. Another idea to reconcile our data with the conventional FS (ref. ^9^) would be to assume a strong temperature dependence of the band parameters such as the effective hopping integral ${\tilde{t}}^{\prime}$ between the next-nearest neighbor Cu sites. In this case, although a good agreement with ∣ ∇ *n*(**k**)∣ is not obtained, one could assume a small $| {\tilde{t}}^{\prime}|$ at 300 K, which then grows to be a large $| {\tilde{t}}^{\prime}|$ at 150 K and decreases at lower temperature, because the conventional FS is measured at 20 K (ref. ^9^) and is fitted to a small $| {\tilde{t}}^{\prime}|$; see Supplementary Note 5 for more details. However, it is not easy to explain such a strong temperature dependence of ${\tilde{t}}^{\prime}$.

In addition, the pseudogap forms below 200 K (ref. ^25^) and is most pronounced around **k** = (*π*, 0) and (0, *π*). Since **k** = (*π*, 0) and (0, *π*) are occupied by electrons for the conventional FS, *n*(**k**) is expected to be suppressed there at 150 K because of a broadening of *n*(**k**); see below and also Supplementary Note 7 for explicit calculations. However, our data Fig. 1c show the opposite, implying that the conventional FS is hard to be reconciled with our data.

### Nematic scenario

As an alternative scenario, we consider a possible effect of nematicity. As the nematicity is observed in other cuprates such as Y-^26–29^ and Bi–based^30^ cuprate compounds, it is natural to invoke the nematicity also in La-based cuprates. As the microscopic origin of the nematicity, several ideas are proposed: fluctuations of charge stripes^31^, a *d*-wave Pomeranchuk instability^32–34^, an orbital order at oxygen sites^35^(The orbital order at oxygen sites is essentially the same as the *d*-wave Pomeranchuk instability, which is obtained as a bond order with wavevector zero and leads to an intra-unit cell order at oxygen sites with wavevector zero), and the nematicity from a biquadratic exchange interaction^36^.

**Fig. 3:**
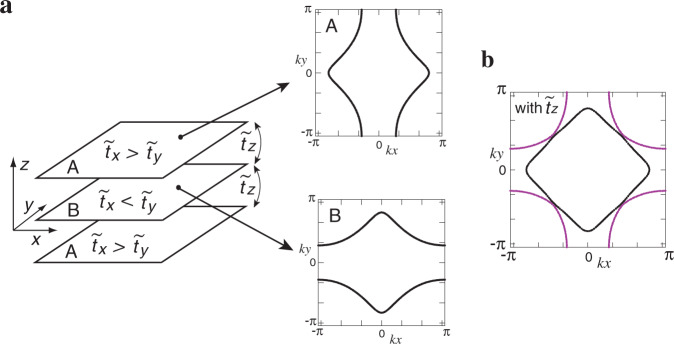
Fermi surface (FS) deformation in La_2−*x*_Sr_*x*_CuO_4_. **a** Alternate stacking of strongly deformed FSs due to the underlaying nematicity in each CuO_2_ plane. ${\tilde{t}}_{x}$ and ${\tilde{t}}_{y}$ are the effective in-plane hopping integrals along the *x* and *y* directions, respectively; ${\tilde{t}}_{z}$ is the effective hopping along the *z* direction; see Supplementary Note 2 for more details. **b** The resulting bulk FSs, which become two-dimensional and consist of inner (black) and outer (purple) FSs.

LSCO with *x* = 0.08 shows the so-called low-temperature orthorhombic (LTO) crystal structure below 350 K (ref. ^37^). This structure is characterized by the lattice anisotropy between [110] and [1$\bar{1}$0] direction, which does not couple to the nematic order. However, there is a low-energy phonon mode with energy <5 meV in the LTO structure^38^. This phonon mode is referred to as the *Z*-point phonon mode and is related to the tilting mode of CuO_6_ octahedra. It yields a dynamical anisotropy between [100] and [010] direction. Consequently, such a *xy* anisotropy couples to the underlying nematic correlations and is strongly enhanced, leading to sizable nematicity. The resulting FS strongly deforms possibly to become a  quasi-one-dimensional  (Q1D) FS, i.e., an open FS. This deformation is expected to occur dynamically with an energy scale of the *Z*-point phonon mode. As the *Z*-point phonon mode has anti-phase correlations along the *z* axis, the Q1D FS is expected to stack alternately along the *z* axis (see Fig. 3a). As a result, bulk FSs consist of two FSs: inner FS and outer FS as shown in Fig. 3b.

The energy scale of Compton scattering is 100 keV much larger than that of the *Z*-point phonon mode. The momentum distribution function observed by Compton scattering, therefore, reflects a snapshot of the alternate stacking of the Q1D FS, especially the most deformed shape where the velocity of the dynamical change of the FS becomes zero. This is indeed a reasonable approximation as we show in Supplementary Note 6.

The idea shown in Fig. 3 was proposed a long time ago theoretically to interpret both magnetic excitation spectra and ARPES date consistently^32,39,40^ and now is supported by our Compton scattering data. Although a Pomeranchuk instability was considered as a physics behind the strong nematicity in early studies^32,33,39,40^, our experimental interpretation does not depend on the microscopic origin of the nematicity.

Furthermore, our model Fig. 3 is applicable to broader situations. First, the atomic pair distribution function analysis of neutron scattering data^41^ suggests a possible local lattice distortion with *xy* anisotropy, which alternates along the *z* axis. This may yield the same stacking pattern as Fig. 3 at least in a certain region around the local lattice distortion. Second, fluctuations associated with the *d*-wave Pomeranchuk instability can have anti-phase correlations between the layers^42^. In this case, our model Fig. 3 is valid even without considering a coupling to other degrees of freedom. On the other hand, the model in Fig. 3 cannot be applicable when there are no anti-phase nematic correlations along the *z* axis. However, as long as nematic correlations are present in each CuO_2_ plane, we expect that the resulting *n*(**k**) can be interpreted in terms of the FSs shown in Fig. 3b. This is because the typical time scale of Compton scattering is much shorter than the electronic one and thus each Compton scattering event detects a snapshot of FS fluctuations and its statistical average is observed as a resulting image. When there are no nematic correlations, our model Fig. 3 is reduced to a usual one where the conventional FS (refs. ^9^ and ^43^) is realized in each CuO_2_ plane.

**Fig. 4:**
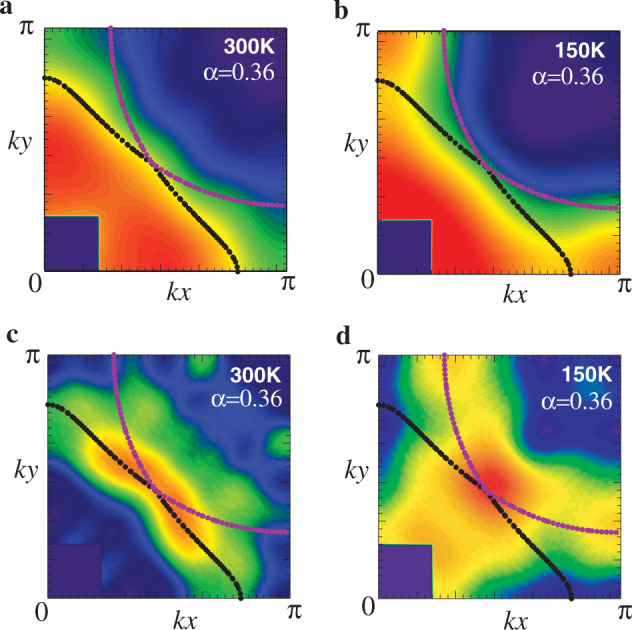
Interpretation of Fig. 1a, b in terms of the nematic scenario shown in Fig. 3. **a** and **c** are the momentum distribution function *n*(**k**) and the magnitude of its derivative ∣ ∇ *n*(**k**)∣ at 300 K in the first quadrant of the Brillouin zone, respectively; **b** and **d** are the corresponding results at 150 K. A value of *α* controls the strength of nematicity and the conventional Fermi surface (ref. ^9^) is reproduced at *α* = 0; see Supplementary Note 2 for the precise definition of *α*.

On the basis of the nematic scenario, we, therefore, invoke the FSs in Fig. 3b and superpose them on our data in Fig. 4a–d. The inner FS at 300 K is located almost perfectly along the constant value of *n*(**k**) (Fig. 4a) and also reasonably traces along the peak position of the gradient of *n*(**k**) (Fig. 4c). For the outer FS, the **k**_*F*_ dependence of *n*(**k**_*F*_) is reasonably small in Fig. 4a. Note that **k**_*F*_ dependence of *n*(**k**_*F*_) around (0.3*π*, 0.9*π*) and (0.9*π*, 0.3*π*) is actually small because the gradient of *n*(**k**) there is very small as seen in Fig. 4c. The peak of ∣ ∇ *n*(**k**)∣ is well captured around (0.45*π*, 0.45*π*) in the red region in Fig. 4c. However, in contrast to the case of the inner FS, it does not extend along the outer FS and the gradient of *n*(**k**) tends to be less pronounced when the momentum goes sufficiently away from (0.45*π*, 0.45*π*). A possible reason is that the occupation number around the outer FS is smaller than that around the inner FS and is closer to the lowest occupation number [dark blue region in Fig. 4a], which may make the gradient of *n*(**k**) less pronounced compared with that around the inner FS.

At 150 K, the outer FS is fully consistent with our data of *n*(**k**) (Fig. 4b) and is located at peak positions of ∣ ∇ *n*(**k**)∣ at **k** = (0.3*π*, 0), (0, 0.3*π*), and (0.45*π*, 0.45*π*) (Fig. 4d). For the inner FS, *n*(**k**) is almost constant along the FS in an extended region around (0.45*π*, 0.45*π*), where ∣ ∇ *n*(**k**)∣ has a peak as expected. Since ∣ ∇ *n*(**k**)∣ becomes very small around **k** = (0, 0.8*π*) and (0.8*π*, 0), a seemingly sizable **k**_*F*_ dependence of *n*(**k**_*F*_) there in Fig. 4b is actually not strong.

**Fig. 5:**
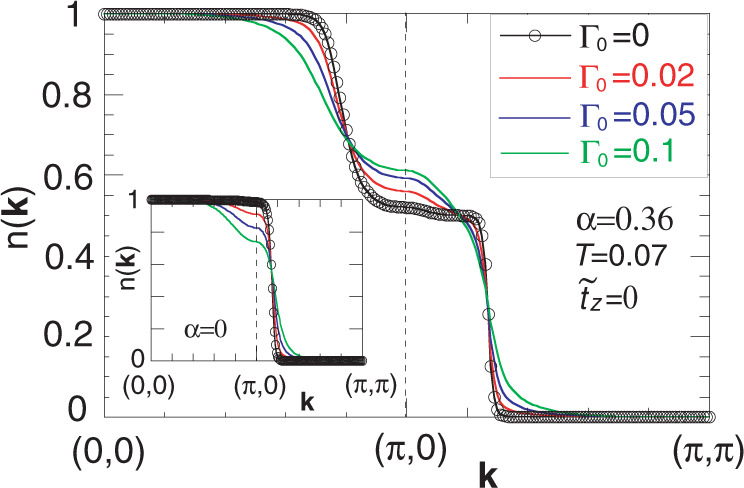
Momentum distribution function *n*(**k**) calculated in the presence of damping of quasiparticles in the nematic scenario presented in Fig. 3. The damping depends on momentum, becomes the largest at (*π*, 0), and its magnitude is controlled by Γ_0_; see Supplementary Note 7 for details. The inset is the corresponding result for the conventional hole-like Fermi surface believed so far for La-based cuprates.

As shown in Fig. 1c, the major difference between 300 K and 150 K appears around **k** = (*π*, 0) and (0, *π*) where *n*(**k**) is enhanced at 150 K, although the pseudogap forms below 200 K (ref. ^25^) and is pronounced at **k** = (*π*, 0) and (0, *π*). This counterintuitive feature is in strong support of the nematic scenario shown in Fig. 3. To demonstrate this, we have performed a phenomenological analysis to model the pseudogap effect in terms of strong damping of quasiparticles especially around **k** = (*π*, 0) and (0, *π*); see Supplementary Note 7 for details. Figure 5 shows *n*(**k**) calculated for several choices of damping Γ_0_ along (0, 0)-(*π*, 0)-(*π*, *π*) direction. Sharp drops at **k** = (0.8*π*, 0) and (*π*, 0.27*π*) correspond to the Fermi momenta. For Γ_0_ = 0, *n*(**k**) is already broad because of the effect of a finite temperature. With increasing the damping, *n*(**k**) is broadened more, leading to an enhancement of *n*(**k**) around **k** = (*π*, 0). This enhancement comes from the asymmetric broadening of *n*(**k**) around the inner and outer FSs. Suppose the typical energy scale of the broadening is Γ_0_, the corresponding broadening of momentum is then estimated as ${{\Delta }}{\bf{k}}={{\Delta }}E\frac{{{\Delta }}{\bf{k}}}{{{\Delta }}E} \sim 2{{{\Gamma }}}_{0}/{{\bf{v}}}_{{\bf{k}}}$ with the velocity **v**_**k**_. For the typical dispersion in cuprates, **v**_**k**_ around **k** = (0.8*π*, 0) becomes much smaller than that around (*π*, 0.27*π*); see Supplementary Eqs. (19, 20). This momentum dependence of **v**_**k**_ is the major reason for the substantial broadening of *n*(**k**) around **k** = (0.8*π*, 0) with the damping of quasiparticles. Consequently, the enhancement of *n*(**k**) occurs around **k** = (*π*, 0).

One may also invoke a gap formation especially around **k** = (*π*, 0) and (0, *π*) to model the pseudogap phenomenology. In this case, Δ*E* corresponds to a gap value, instead of a damping Γ_0_, and we still obtain results similar to Fig. 5. The microscopic origin to yield Δ*E*, namely the origin of the pseudogap itself, remains elusive and is beyond the scope of the present measurements.

At both 300 K and 150 K (Fig. 4c, d), the intensity becomes strong in an extended region around (0.45*π*, 0.45*π*), where the inner and outer FSs are close to each other. This kind of feature is also observed in Sr_2_RuO_4_ (ref. ^13^), where three FSs nearly intersect around (2*π*/3, 2*π*/3) and the intensity of the first derivative of *n*(**k**) becomes strongest there in a wide region. The extended region of strong intensity at 300 K (Fig. 4c) shrinks and concentrates around **k** = (0.45*π*, 0.45*π*) at 150 K (Fig. 4d). This is reasonable because the effect of the pseudogap is weak around **k** = (0.45*π*, 0.45*π*) and the sharpest feature of ∣ ∇ *n*(**k**)∣ should be observed there.

**Fig. 6:**
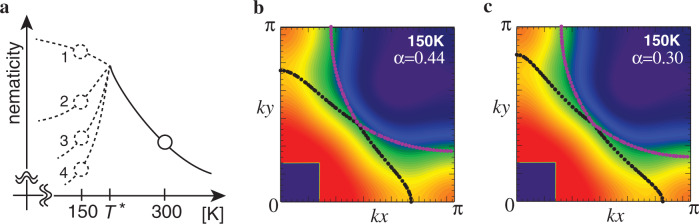
Possible temperature dependence of nematicity. **a** Sketch of temperature dependence of the nematicity. The pseudogap temperature T^*^ is estimated around 200 K (ref. ^25^). Given that the system is close to a nematic instability, the nematicity is expected to increase with decreasing temperature at least down to T^*^. It is not clear how the nematicity develops below T^*^ and thus four possible scenarios are shown: the nematicity increases (scenario 1) or decreases (scenarios 2–4). In the latter case, compared with the nematicity at 300 K, the nematicity at 150 K becomes larger (scenario 2), comparable (scenario 3), or smaller (scenario 4). **b** and **c** are typical Fermi surfaces for scenarios 1 and 2, and scenario 4, respectively; they are superposed on the observed map of *n*(**k**) at 150 K. The value of *α* is a parameter of the nematicity and the conventional Fermi surface (ref. ^9^) is reproduced for *α* = 0. See Supplementary Fig. 8 for a comparison with the gradient of *n*(**k**).

Because the large anisotropy comes from the underlying nematic correlations in the electron system, the anisotropy is expected to have a strong temperature dependence as indicated theoretically^33,36^. As the so-called pseudogap temperature T^*^ is  around 200 K for *x* = 0.08 (ref. ^25^) and the gap-like feature develops around **k** = (*π*, 0) and (0, *π*) below T^*^, the temperature dependence of the nematicity likely changes below T^*^. As sketched in Fig. 6a, there are two possibilities below T^*^: the nematicity still develops or is suppressed. In the latter case, it is not clear whether the nematicity at 150 K becomes larger or smaller than that at 300 K or comparable to that. Figures 4b, 6b, and c indicate that our data at 150 K is equally well fitted by FSs with different degrees of the nematicity; see Supplementary Note 8 for more details. This ambiguity comes from a rather broad feature of *n*(**k**) and its derivative, which is mainly owing to strong electron correlations typical to cuprate superconductors. It remains to be studied which scenario shown in Fig. 6a is likely appropriate to LSCO. In addition, the determination of the temperature, at which the nematicity becomes sizable, is also left to a future study.

## Discussion

The present Compton scattering experiments provide three important insights into the electronic property in cuprate superconductors.

First, our observed images for La-based cuprates imply that the FS is strongly deformed by the underlying nematicity in each CuO_2_ plane, but the bulk FSs recover the fourfold symmetry. The resulting electronic property does not exhibit anisotropy in bulk in spite of the strong nematicity. This is in total contrast to Y-based cuprates, where the intrinsic small *xy* anisotropy is strongly enhanced as observed by resistivity^26^, neutron scattering^27^, Nernst coefficient^28^, and magnetic torque^29^.

Second, the momentum distribution function is enhanced around **k** = (*π*, 0) and (0, *π*) in the pseudogap phase, although the pseudogap itself is most pronounced there. This counterintuitive feature implies the presence of the reconstructed FSs owing to the underlying nematicity.

Third, our observed images cannot be reconciled with the conventional hole-like FS (ref. ^9^). Nonetheless, our proposed FSs are not inconsistent with the ARPES spectra. ARPES is most precise around the so-called nodal region for cuprates. In fact, the position of our FSs near **k** = (0.45*π*, 0.45*π*) almost coincides with the FS from ARPES (ref. ^9^); compare Fig. 4 with Fig. 2. Near **k** = (0, *π*), on the other hand, the ARPES data in refs. ^43,44^ show a broad peak, which was interpreted as a single peak. Such a broad peak may also be interpreted as consisting of the underlying double-peak structure originating from the inner and outer FSs as implied from the present Compton scattering experiments. The complementary employment of ARPES and Compton scattering will provide more-detailed information to elucidate the electronic structure in cuprates.

We have focused on the underdoped LSCO with *x* = 0.08. It is natural to ask how the nematicity and the shape of the FSs evolve with increasing doping. At a fixed temperature, nematic correlations are expected to be less pronounced with increasing doping rate and a conventional FS may be realized eventually in the heavily overdoped region. By performing Compton scattering measurements for LSCO with *x* = 0.15 and 0.30 at 300 K, we have confirmed that the data at *x* = 0.15 can be understood with weaker nematicity than *x* = 0.08, whereas those at *x* = 0.30 imply a conventional electron-like FS but possibly tiny nematicity cannot be excluded. Details are given in Supplementary Note 11.

FS deformation associated with nematicity is also observed in Fe-based superconductors^45^. Its effect is, however, not so drastic as the one that we have found in La-based cuprates. This is because the FS in cuprates is located close to **k** = (*π*, 0) and (0, *π*), and thus the nematicity can change easily the FS topology.

Finally, the present experiments indicate that Compton scattering can be a powerful tool to elucidate the FS and work beyond the widely employed techniques such as ARPES and quantum oscillations. As Compton scattering is neither disorder nor surface sensitive and is available also in the presence of electric and magnetic fields, it will be exciting that Compton scattering is employed as a complementary tool to ARPES and quantum oscillations in various correlated electron systems.

## Methods

High-resolution Compton scattering requires a large single crystal to map the momentum distribution function. Our high-quality single crystals of LSCO with *x* = 0.08, 0.15, and 0.30 were grown by a traveling-solvent floating-zone method. The feed rod was prepared by the solid-state reaction from La_2_O_3_, SrCO_3_, and CuO in the molar ratio of (2 − *x*)/2: *x*: 1.0. The mixed powder was sintered at ~ 1000 °C for 24 hours in air with intermediate grinding. The sintered powder was then pressed into the cylindrical rod and again sintered at 1250 °C for 24 hours in air. We used this feed rod as well as a solvent with a composition of La_2−*x*_Sr_*x*_CuO_4_:CuO_2_ = 1:4.7 and La_2_CuO_4_ as a seed rod for the crystal growth in an infrared radiation furnace with oxygen gas flow rate of 100 cm^3^/min. The growth rate was set to be 1.0 mm/h. We obtained a 100 mm-long crystal rod and annealed it in oxygen gas flow to minimize oxygen deficiencies. A part of the grown rod was cut into the cubic-shaped portion about the size of 5 × 5 × 5 mm^3^ for our Compton measurements. We measured the magnetic susceptibility and confirmed the superconducting transition with *T*_*c*_ ≈ 20 K, 38 K, and 0 K at *x* = 0.08, 0.15, and 0.30, respectively, from the Meissner signal.

We measured Compton profiles with scattering vectors equally spaced between the [100] and [110] directions using the Cauchois-type x-ray spectrometer at the BL08W beamline of SPring-8. The overall momentum resolution is estimated to be 0.13 a.u. in FWHM. The incident x-ray energy was 115 keV and the scattering angle was 165 degrees. The Compton-scattered x-rays were measured by a two-dimensional position-sensitive detector. The energy distribution of the Compton-scattered x-rays was centered at about 80 keV. Approximately 3 × 10^5^ counts in total were collected at the Compton peak channel. Each Compton profile was corrected for absorption, analyzer, detector efficiencies, scattering cross-section, possible double scattering contributions, and x-ray background^46^. A two-dimensional momentum density, representing a projection of the three-dimensional momentum density onto the *ab* plane (see Supplementary Note 9 for the effect of *k*_*z*_ dispersion), was reconstructed from each set of ten Compton profiles by using the direct Fourier transform method^47^. This method produces unphysical oscillations at low momenta, which is the reason why there occur large errors in the central area of the experimental *n*(**k**). To fold the obtained *n*(**k**) into a single central Brillouin zone, we employ the LCW theorem^24^. This theorem is exact for non-interacting electrons, but an approximation for interacting electrons.

## Supplementary information

Supplementary Information

Peer Review File

## Data Availability

The data that support the finding of this study are available from H.Y. and Y.S. upon reasonable request.
