# Supplementary material for: Fermi surface in La-based cuprate superconductors from Compton scattering imaging
Source: Nat Commun. 2021 Apr 13;12:2223. doi: 10.1038/s41467-021-22229-6 (PMC8044246; doi:10.1038/s41467-021-22229-6)
Supplement: Supplementary file 1 — Supplementary Information [file 41467_2021_22229_MOESM1_ESM.pdf]

Supplementary Information:
Fermi surface in La-based cuprate superconductors from Compton scattering imaging

Hiroyuki Yamase[1,2*], Yoshiharu Sakurai[3], Masaki Fujita[4], Shuichi Wakimoto[5], and Kazuyoshi Yamada[6]

[1]International Center for Materials Nanoarchitectonics, National Institute for Materials Science (NIMS), Tsukuba 305-0047, Japan

[2]Department of Condensed Matter Physics, Graduate School of Science, Hokkaido University, Sapporo 060-0810, Japan

[3]Japan Synchrotron Radiation Research Institute (JASRI), SPring-8, Hyogo 679-5198, Japan

[4]Institute for Materials Research, Tohoku University, Sendai 980-8577, Japan

[5]Materials Sciences Research Center, Japan Atomic Energy Agency, Tokai, Naka, Ibaraki 319-1195, Japan

[6] High Energy Accelerator Research Organization (KEK), Tsukuba 305-0801, Japan

Supplementary Note 1: General features of $n(\mathbf{k})$

The momentum distribution function $n(\mathbf{k})$ is a central quantity in the present work. Here we recall its general feature. In a noninteracting electron system (Supplementary Fig. 1a), $n(\mathbf{k}) = 1$ in $\mathbf{k} < \mathbf{k}_F$, 0.5 at $\mathbf{k} = \mathbf{k}_F$, and 0 in $\mathbf{k} < \mathbf{k}_F$ (Ref. 1); here $\mathbf{k}_F$ is Fermi momentum. In a Fermi liquid (Supplementary Fig. 1b), $n(\mathbf{k})$ is similar to the noninteracting case, but the magnitude of a jump of $n(\mathbf{k})$ at $\mathbf{k} = \mathbf{k}_F$ is reduced to $Z_F < 1$. Still $n(\mathbf{k}_F)$ is expected around 0.5; it is not necessarily to be exactly 0.5. In a non-Fermi liquid state (Supplementary Fig. 1c) such as a Tomonaga-Luttinger liquid well established in a one-dimensional system, $n(\mathbf{k})$ exhibits power-law behavior around $\mathbf{k}_F$ and there is no jump at $\mathbf{k}_F$. A value of $n(\mathbf{k}_F)$ is usually around 0.5. In the limit of strong correlations where double occupancy of electrons is forbidden (Supplementary Fig. 1d), a general insight can be obtained. Suppose hole carriers are introduced into the half-filled antiferromagnetic Mott insulator. When the carrier density is δ, we obtain the sum rule[2]

$$\int_{-\infty}^{\infty} A(\mathbf{k},\omega)\mathrm{d}\omega = \frac{1}{2}(1+\delta)\,. \tag{1}$$

Hence it follows that for any $\mathbf{k}$ point

$$n(\mathbf{k}) = \int_{-\infty}^{\infty} A(\mathbf{k},\omega) f(\omega)\mathrm{d}\omega \leq \frac{1}{2}(1+\delta)\,. \tag{2}$$

Supplementary Eqs. (1) and (2) are exact relations. At $\delta = 0$ where one electron resides at each site, the electron density is given by $\frac{2}{N}\sum_{\mathbf{k}} n(\mathbf{k}) = 1$, where N is the total number of lattice sites and the factor of 2 counts the spin degrees of freedom. Supplementary Eq. (2) then implies $n(\mathbf{k}) = 0.5$ independent of $\mathbf{k}$ at $\delta = 0$. When carriers are doped and the system becomes metallic, we have the FS. We then expect $n(\mathbf{k}) > n(\mathbf{k}_F)$ and $n(\mathbf{k}) < n(\mathbf{k}_F)$ for $\mathbf{k}$ inside and outside the FS, respectively, and $n(\mathbf{k}_F) \sim 0.5$ on the FS, as indeed found in a variational Monte Carlo study in the t-J model[3,4].

Supplementary Note 2: Formalism of momentum distribution function

It is insightful to see how the momentum distribution function looks in the simplest case for a realistic layered system of La-based cuprates where the unit cell contains two different layers and each layer shifts by $[\frac{1}{2}, \frac{1}{2}, \frac{1}{2}]$ (Ref. 5). We denote each layer by the A and B plane

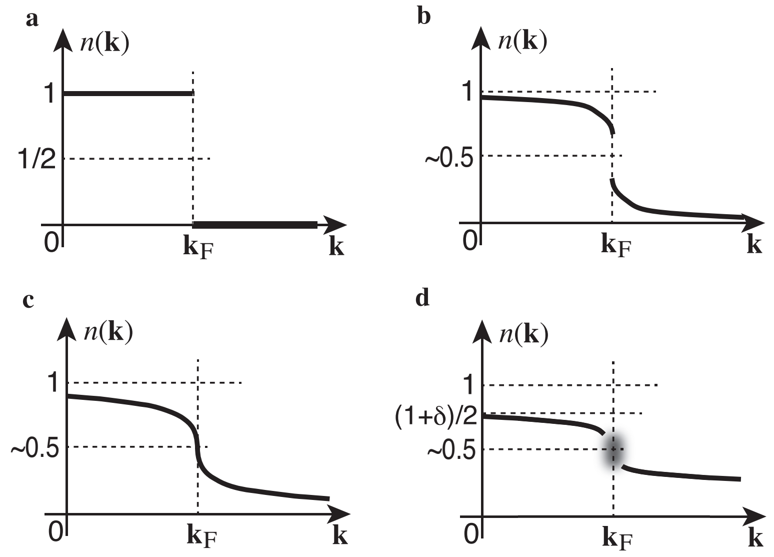

Supplemenatary Fig. 1. Sketches of the momentum distribution function at zero temperature in typical cases: (**a**) non-interaction system, (**b**) Fermi liquid system, (**c**) non-Fermi liquid system such as a Tomonaga-Luttinger liquid, and (**d**) the limit of the strong correlation. It is not clear whether $n(\mathbf{k})$ exhibits a jump or a smooth evolution around the Fermi momentum $\mathbf{k}_F$ in (**d**).

as shown in Fig. 3a and we consider the following minimal one-body Hamiltonian:

$$\tilde{\mathcal{H}} = \sum_{\mathbf{k}\sigma} \begin{pmatrix} c^{A\dagger}_{\mathbf{k}\sigma} & c^{B\dagger}_{\mathbf{k}\sigma} \end{pmatrix} \begin{pmatrix} \xi^A_{\mathbf{k}} & \epsilon_{\mathbf{k}} \\ \epsilon_{\mathbf{k}} & \xi^B_{\mathbf{k}} \end{pmatrix} \begin{pmatrix} c^A_{\mathbf{k}\sigma} \\ c^B_{\mathbf{k}\sigma} \end{pmatrix} . \tag{3}$$

Here $c^{A\dagger}_{\mathbf{k}\sigma}(c^A_{\mathbf{k}\sigma})$ is the creation (annihilation) operator of electrons with momentum $\mathbf{k}$ and spin σ in the A plane, $\xi^A_{\mathbf{k}}$ the in-plane dispersion, and $\epsilon_{\mathbf{k}}$ the c-axis dispersion. Similarly the corresponding quantities are defined for the B plane. This one-body Hamiltonian can be regarded as an effective Hamiltonian and may be obtained after a mean-field approximation to an interacting electron system. It is straightforward to compute the eigenenergies of the Hamiltonian:

$$\lambda_\pm(\mathbf{k}) = \frac{\xi^A_{\mathbf{k}} + \xi^B_{\mathbf{k}}}{2} \pm \sqrt{\left(\frac{\xi^A_{\mathbf{k}} - \xi^B_{\mathbf{k}}}{2}\right)^2 + \epsilon^2_{\mathbf{k}}} \, . \tag{4}$$

We normalize the momentum distribution function $n(\mathbf{k})$ in a way that its maximal value is unity:

$$n(\mathbf{k}) = \frac{1}{4} \sum_{\sigma} \left(\left\langle c^{A\dagger}_{\mathbf{k}\sigma} c^A_{\mathbf{k}\sigma} \right\rangle + \left\langle c^{B\dagger}_{\mathbf{k}\sigma} c^B_{\mathbf{k}\sigma} \right\rangle \right) . \tag{5}$$

Using the Fermi distribution function $f(\lambda) = 1/(\mathrm{e}^{\lambda/T} + 1)$, where T is temperature, the

right-hand side of the above equation is easily computed as

$$n(\mathbf{k}) = \frac{1}{2}\int_{-\infty}^{\infty} d\omega \left(A_+(\mathbf{k},\omega) + A_-(\mathbf{k},\omega)\right) f(\omega)\,, \tag{6}$$

$$= \frac{1}{2}\left(1 - \frac{1}{2}\tanh\frac{\lambda_+(\mathbf{k})}{2T} - \frac{1}{2}\tanh\frac{\lambda_-(\mathbf{k})}{2T}\right)\,. \tag{7}$$

Here we have introduced the spectral function $A_\pm(\mathbf{k},\omega)$ for a later convenience, which is given by $A_\pm(\mathbf{k},\omega) = \delta(\omega - \lambda_\pm(\mathbf{k}))$ in the present case. When $\xi_{\mathbf{k}} = \xi^A_{\mathbf{k}} = \xi^B_{\mathbf{k}}$ and $\epsilon_{\mathbf{k}} = 0$, the above expression is reduced to the well-known Fermi distribution function

$$n(\mathbf{k}) = \frac{1}{2}\left(1 - \tanh\frac{\xi_{\mathbf{k}}}{2T}\right)\,. \tag{8}$$

Since the CuO_2 plane forms a square lattice and we will allow xy anisotropy of the band dispersion, we parameterize the in-plane dispersion as

$$\xi^A_{\mathbf{k}} = -2\left(\tilde{t}_x \cos k_x + \tilde{t}_y \cos k_y\right) - 4\tilde{t}^{'} \cos k_x \cos k_y - 2\left(\tilde{t}^{''}_x \cos 2k_x + \tilde{t}^{''}_y \cos 2k_y\right) - \mu \tag{9}$$

where $\tilde{t}_x = \tilde{t}(1+\frac{1}{2}\alpha)$, $\tilde{t}_y = \tilde{t}(1-\frac{1}{2}\alpha)$, $\tilde{t}^{''}_x = \tilde{t}^{''}(1+\frac{1}{2}\alpha'')$, $\tilde{t}^{''}_y = \tilde{t}^{''}(1-\frac{1}{2}\alpha'')$, and μ is the chemical potential. Because of possible coupling to the Z-point phonons[6], the in-plane dispersion in the adjacent plane may have the opposite anisotropy, namely

$$\xi^B_{\mathbf{k}} = -2\left(\tilde{t}_y \cos k_x + \tilde{t}_x \cos k_y\right) - 4\tilde{t}^{'} \cos k_x \cos k_y - 2\left(\tilde{t}^{''}_y \cos 2k_x + \tilde{t}^{''}_x \cos 2k_y\right) - \mu\,. \tag{10}$$

The A and B planes are shifted by $[\frac{1}{2}, \frac{1}{2}, \frac{1}{2}]$ with each other. The c-axis dispersion may be parameterized by

$$\epsilon_{\mathbf{k}} = -8\tilde{t}_z \cos\frac{k_x}{2}\cos\frac{k_y}{2}\cos\frac{k_z}{2}\,. \tag{11}$$

The angle-resolved photoemission spectroscopy (ARPES) study[7] suggests a different form

$$\epsilon_{\mathbf{k}} = -2\tilde{t}_z \cos\frac{k_x}{2}\cos\frac{k_y}{2}\cos\frac{k_z}{2}\left[(\cos k_x - \cos k_y)^2 + c_0\right]\,, \tag{12}$$

with $c_0 = 0$. This functional form shall also be considered.

We first take $\alpha = \alpha'' = 0$ and $\tilde{t}_z = 0$ and fit the FS to the ARPES data at $x = 0.07$ (Ref. 8) under the condition $\tilde{t}^{''} = -\tilde{t}^{'}/2$ (Ref. 9), which yields the following values:

$$\tilde{t}^{'} = -0.15\tilde{t}, \quad \tilde{t}^{''} = 0.075\tilde{t} \quad \text{for } x = 0.07\,. \tag{13}$$

At $x = 0.22$, $\tilde{t}_z$ is estimated around $0.07\tilde{t}$ in the analysis of the ARPES data at 12 K (Ref. 7). The coherency along the z direction is best achieved in the superconducting state and likely

becomes worse with increasing temperature. In fact, the c-axis resistivity is non-metallic in the state above T_c (Ref. 10). Hence in our measurements at 300 K and 150 K a value of $\tilde{t}_z$ may be smaller than $\tilde{t}_z = 0.07\tilde{t}$. In addition, because of the strong correlation effect specific to cuprate superconductors, the effective value of $\tilde{t}_z$ is proportional to the carrier density as indeed the case of the t-J model[11]. This effect also works to suppress a value of $\tilde{t}_z$ at $x = 0.08$. Given a likely small value of $\tilde{t}_z$ (see the Supplementary Note 10 for possible values of $\tilde{t}_z$), it turns out that our conclusions obtained in the present work do not depend on a precise choice of $\tilde{t}_z$ nor on a choice of the c-axis dispersions [Supplementary Eqs. (11) and (12)]. Hence to keep our presentation as simple as possible, we took $\tilde{t}_z = 0$ to compute the FSs in Figs. 4 and 6.

We measure all quantities with the dimension of energy in units of $\tilde{t}$. Our parameters α and α'' determine a band anisotropy and we assume $\alpha = \alpha''$ for simplicity. The chemical potential μ is determined to reproduce the doping rate. $n(\mathbf{k})$ depends on not only in-plane momentum but also out-of-plane momentum. Since our Compton scattering data integrate k_z dependence, we consider

$$n(k_x, k_y) = \frac{1}{N_z} \sum_{k_z} n(\mathbf{k}) \tag{14}$$

where N_z is a half of the number of the layers, namely the number of the unit cell along the z direction. We took $N_z = 48$, which is sufficiently large. To keep the same notation as the main text we write $n(k_x, k_y)$ as $n(\mathbf{k})$ below.

Supplementary Note 3: Fermi surfaces

The FS is obtained by solving $\lambda_\pm(\mathbf{k}) = 0$ in Supplementary Eq. (4) and always fulfills Luttinger's theorem. Since the unit cell contains two layers, we generally obtain two FSs for each k_z. In principle, the FS depends on temperature via the chemical potential μ. However, such an effect is negligible in a temperature range that we are interested in. Rather, as we discussed in Fig. 6a, the sizable temperature dependence is expected for the parameter α introduced in the band dispersions [see Supplementary Eqs. (9) and (10)] due to the underlying nematic correlations.

Supplementary Note 4: Thermal broadening effect

To see the thermal broadening effect on $n(\mathbf{k})$, we compute $n(\mathbf{k})$ for $\alpha = 0$ and $\tilde{t}_z = 0$.

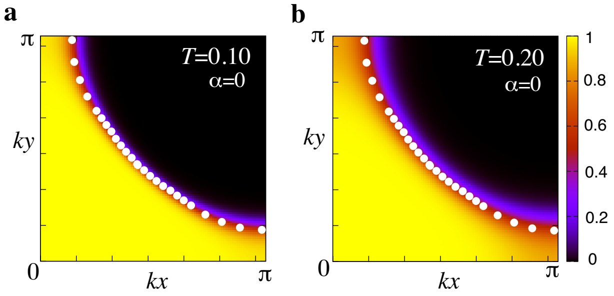

Supplemenatary Fig. 2. Momentum distribution function $n(\mathbf{k})$ of the Hamiltonian (3) at $T = 0.1$ (**a**) and 0.2 (**b**). The band parameters are chosen to reproduce the Fermi surface reported in the angle-resolved photoemission spectroscopy measurements (white circles)[8]; see Supplementary Eq. (13).

Supplementary Fig. 2 shows the results in the first quadrant of the Brillouin zone at $T = 0.1$ and 0.2, which may be associated with the data at 150 K and 300 K, respectively. We superpose the FS reported by ARPES (Ref. 8) on Supplementary Fig. 2. Since electron correlation effects are considered only via the effective one-body Hamiltonian in the present calculations [see Supplementary Eq. (3)], $n(\mathbf{k})$ shows a sharp drop at the Fermi momentum $\mathbf{k}_F$. With increasing T, $n(\mathbf{k})$ is broadened around $\mathbf{k}_F$, but we see no signature that $n(\mathbf{k}_F)$ starts to depend on $\mathbf{k}_F$ by thermal broadening.

It is apparent that as long as we stick to the conventional FS, the map of $n(\mathbf{k})$ shown in Supplementary Fig. 2 is hard to be reconciled with our data (Fig. 2a, b). In fact, a comparison with Fig. 2 implies a possible internal structure in the yellow region in Supplementary Fig. 2. Our idea of the nematicity actually produces an inner FS as we discussed in Fig. 3.

Supplementary Note 5: Possible cure of the conventional Fermi surface

It is difficult to reconcile the conventional FS reported by ARPES (Ref. 8) with our data (Fig. 2) as we discussed in Results section as well as Supplementary Note 4. A possible cure of this conventional idea may be to introduce a temperature dependence of the band parameter $\tilde{t}'$ under the condition $\tilde{t}'' = -\tilde{t}'/2$ (Ref. 9). We would then assume around $\tilde{t}' = -0.08$ at 300 K and $\tilde{t}' = -0.24$ at 150 K so that $n(\mathbf{k}_F)$ has a weak $\mathbf{k}_F$ dependence (Supplementary Fig. 3a, b), although the agreement with the map of $|\nabla n(\mathbf{k})|$ is not so satisfactory in the sense that the curvature around $\mathbf{k} = (0.45\pi, 0.45\pi)$ is different at 300 K

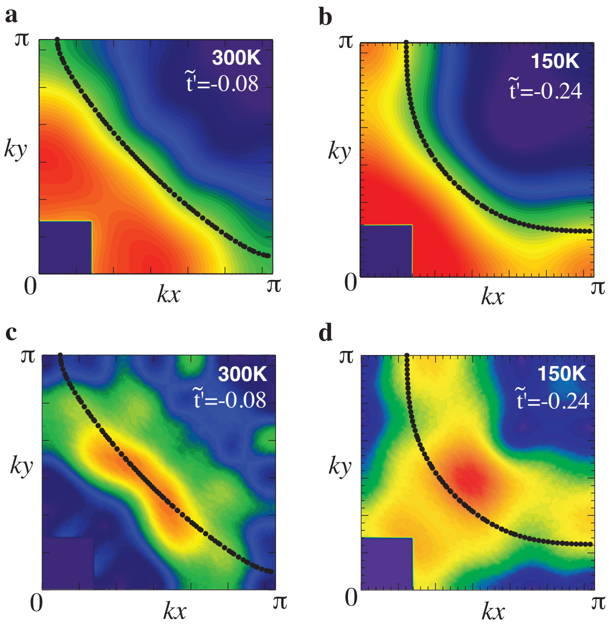

Supplemenatary Fig. 3. Fit to our data (Fig. 1) in terms of a conventional Fermi surface, namely without nematicity ($\alpha = 0$) at 300 K (**a**) and 150 K (**b**). The band parameter $\tilde{t}'$ is taken as $\tilde{t}' = -0.08$ and -0.24 at 300 K and 150 K, respectively. (**c**) and (**d**) the corresponding maps of a derivative of the momentum distribution function $n(\mathbf{k})$.

(Supplementary Fig. 3c) and the conventional FS is away from the peak position of $|\nabla n(\mathbf{k})|$ at $(0.3\pi, \pi)$ and $(\pi, 0.3\pi)$ at 150 K (Supplementary Fig. 3d). In addition, the FS seems to deviate sufficiently from the strongest peak of $|\nabla n(\mathbf{k})|$ around $\mathbf{k} = (0.45\pi, 0.45\pi)$ at 150 K in Supplementary Fig. 3d. Recalling that ARPES data[8] was taken at 20 K and the reported FS is well fitted with $\tilde{t}' = -0.15$ there when we stick to the conventional FS [see Supplementary Eq. (13)], we would need to assume a strong and non-monotonous temperature dependence of $\tilde{t}'$ between 300 K and 20 K, which does not seem realistic.

Supplementary Note 6: Time averaging effect of FS fluctuations

We consider a coupling to the Z-point phonon mode[6]. In this case, the anisotropy of the FS is expected to be fluctuating in time with the same scale as the phonon, which is less than 5 meV. On the other hand, Compton scattering is a high-energy probe and observes a snapshot of the fluctuating FS at each time. The resulting signal is time-averaged. We

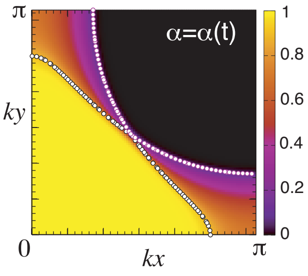

Supplemenatary Fig. 4. Time-averaged momentum distribution function $\bar{n}(\mathbf{k})$ for $\alpha_{\max} = 0.36$ [Supplementary Eqs. (15) and (16)] at temperature $T = 0.07$ and interlayer hopping $\tilde{t}_z = 0$. The Fermi surface for $\alpha = \alpha_{\max}$ is superposed (circles).

model the time-dependent anisotropy of the FS as

$$\alpha(t) = \alpha_{\max} \cos(\omega_Z t + \phi) \tag{15}$$

where ω_Z is the frequency of the Z-point phonons, t time, and ϕ a phase. We may write the momentum distribution function $n(\mathbf{k})$ as $n(\mathbf{k}, \alpha(t))$. The time-averaged momentum distribution function $\bar{n}(\mathbf{k})$ is then given as

$$\bar{n}(\mathbf{k}) = \frac{1}{T_Z} \int_0^{T_Z} n(\mathbf{k}, \alpha(t)) \mathrm{d}t \,, \tag{16}$$

where $T_Z = 2\pi/\omega_Z$ is the periodicity in time. $\bar{n}(\mathbf{k})$ for $\alpha_{\max} = 0.36$ is shown in Supplementary Fig. 4. While $\bar{n}(\mathbf{k})$ exhibits a broadened feature, it is well characterized by the FS with $\alpha = \alpha_{\max}$.

Supplementary Note 7: Phenomenological study of a pseudogap effect

Here we present a full description of a phenomenological study of a pseudogap effect on $n(\mathbf{k})$ in terms of a quasiparticle damping; the essential part is already given in the main text.

As a minimal model, we replace the spectral function in Supplementary Eq. (6) as

$$A_{\pm}(\mathbf{k}, \omega) = \frac{1}{\pi} \frac{\Gamma_{\mathbf{k}}}{(\omega - \lambda_{\pm}(\mathbf{k}))^2 + \Gamma_{\mathbf{k}}^2} \,, \tag{17}$$

where $\Gamma_{\mathbf{k}}$ represents a damping of quasiparticles. Since the pseudogap effect is most pronounced around $\mathbf{k} = (\pi, 0)$ and $(0, \pi)$ (Ref. 12), we assume the following $\mathbf{k}$ dependence:

$$\Gamma_{\mathbf{k}} = \Gamma_0 (\cos k_x - \cos k_y)^2 + T^2 \,. \tag{18}$$

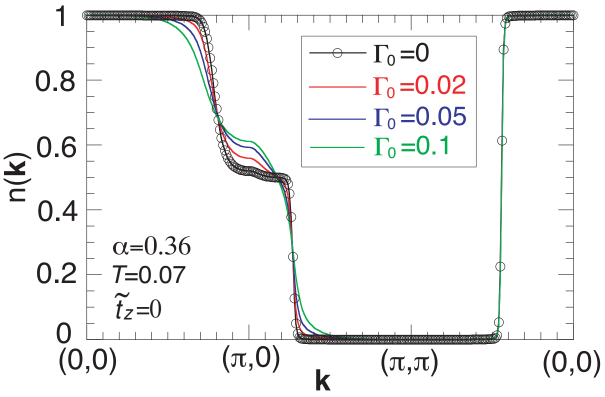

Supplemenatary Fig. 5. Momentum distribution function $n(\mathbf{k})$ of the Hamiltonian (3) for nematicity $\alpha = 0.36$ at temperature $T = 0.07$ and doping $\delta = 0.08$. Strength of quasiparticle damping is parameterized by Γ_0 in Supplementary Eq. (18).

The second term is a regular contribution and relevant only around the nodal direction where the first term vanishes. We compute $n(\mathbf{k})$ under the condition of charge conservation, namely $n = \frac{1}{N}\sum_{\mathbf{k}\sigma} n(\mathbf{k}) = 1 - \delta$, where N is the total number of the lattice cites. We neglect the effect of c-axis dispersion, which is irrelevant to the present analysis,

In Supplementary Fig. 5 we plot $n(\mathbf{k})$ along the symmetry axis for several choices of Γ_0 at $T = 0.07$. $n(\mathbf{k})$ shows rapid changes at $\mathbf{k} = (0.8\pi, 0)$, $(\pi, 0.27\pi)$, and $(0.45\pi, 0, 45\pi)$, which correspond to the Fermi momenta for $\alpha = 0.36$. The sharp feature around $(0.45\pi, 0, 45\pi)$ stays even with increasing Γ_0, because the d-wave form factor in Supplementary Eq. (18) vanishes. On the other hand, $n(\mathbf{k})$ is broadened with Γ_0 around $(0.8\pi, 0)$ and $(\pi, 0.27\pi)$ and the broadening is more pronounced around $(0.8\pi, 0)$. This asymmetry originates from the typical band structure of cuprates. The quasiparticle dispersions [Supplementary Eq. (4)] are given by $\xi^A_{\mathbf{k}}$ [Supplementary Eq. (9)] and $\xi^B_{\mathbf{k}}$ [Supplementary Eq. (10)] when we neglect the c-axis dispersion. $\xi^A_{\mathbf{k}}$ crosses the FS around $\mathbf{k} = (0.8\pi, 0)$, whereas $\xi^B_{\mathbf{k}}$ does around $\mathbf{k} = (\pi, 0.27\pi)$. The velocity of $\xi^A_{\mathbf{k}}$ at $\mathbf{k} = (k_x, 0)$ is given by

$$\mathbf{v}^A_{\mathbf{k}} = \left(2(\tilde{t}_x + 2\tilde{t}' + 4\tilde{t}''_x \cos k_x) \sin k_x, 0\right) , \tag{19}$$

and that of $\xi^B_{\mathbf{k}}$ along $\mathbf{k} = (\pi, k_y)$ is

$$\mathbf{v}^B_{\mathbf{k}} = \left(0, 2(\tilde{t}_x - 2\tilde{t}' + 4\tilde{t}''_x \cos k_y) \sin k_y\right) . \tag{20}$$

We obtain $|\mathbf{v}^A_{\mathbf{k}}| = 0.69$ at $\mathbf{k} = (0.8\pi, 0)$ and $|\mathbf{v}^B_{\mathbf{k}}| = 2.57$ at $\mathbf{k} = (\pi, 0.27\pi)$ for the present parameters. This big difference comes from the presence of $\tilde{t}'$ and $\tilde{t}''$. Since the quasiparticle

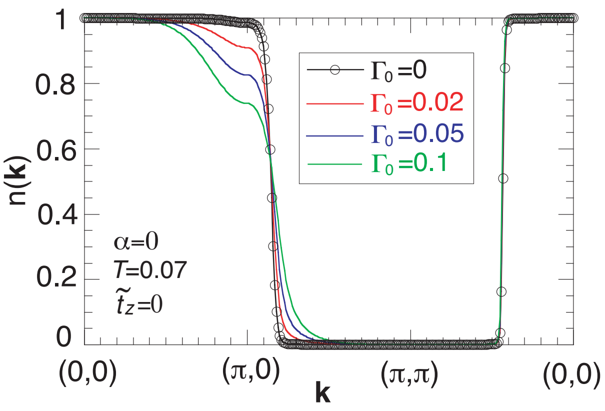

Supplemenatary Fig. 6. Momentum distribution function $n(\mathbf{k})$ of the Hamiltonian (3) without nematicity ($\alpha = 0$) at temperature $T = 0.07$ and doping $\delta = 0.08$. Strength of quasiparticle damping is parameterized by Γ_0 in Supplementary Eq. (18).

damping $\Gamma_{\mathbf{k}}$ gives rise to the broadening of momentum as $\Delta\mathbf{k} = \Delta E \frac{\Delta\mathbf{k}}{\Delta E} \sim 2\Gamma_{\mathbf{k}}/\mathbf{v}_{\mathbf{k}}$, the momentum distribution function $n(\mathbf{k})$ becomes much broader around $\mathbf{k} = (0.8\pi, 0)$ than $(\pi, 0.27\pi)$. In addition, compared with the result for $\Gamma_0 = 0$, this broadening gives rise to an increase of the occupation around $\mathbf{k} = (\pi, 0)$, although the damping $\Gamma_{\mathbf{k}}$ is biggest there. This unexpected feature comes from the presence of two Fermi momenta around $\mathbf{k} = (\pi, 0)$, typical to the nematic scenario (Fig. 3).

Since the pseudogap forms below 200 K and its maximal energy scale is around 40 meV (Ref. 13), we may associate the results for $\Gamma_0 \approx 0.05 - 0.1 (\approx 0 - 0.01)$ to our data at 150 K (300 K), considering $\tilde{t} \sim 100$ meV. This phenomenological analysis explains i) $n(\mathbf{k})$ is broadened around $\mathbf{k} = (0.8\pi, 0)$ substantially more than around $\mathbf{k} = (\pi, 0.27\pi)$ at 150 K as observed in Fig. 4b, d, ii) the resulting signal is enhanced around $\mathbf{k} = (\pi, 0)$ and $(0, \pi)$ compared with the data at 300 K (see Fig. 1c), and iii) the region around $\mathbf{k} = (0.45\pi, 0.45\pi)$ stays essentially the same at both 300 K and 150 K (see Fig. 4c, d).

In the present simple analysis, we have introduced the damping of the Lorentzian form [Supplementary Eq. (17)] into the effective Hamiltonian (3). Hence the maximum and minimum values of $n(\mathbf{k})$ become 1 and 0, respectively. Physically, however, in cuprates we would expect the maximum value of $n(\mathbf{k})$ is around $(1 + \delta)/2$ [see Supplementary Eq. (2)] and the minimum value is well above zero as seen in variational Monte Carlo study in the t-J model[4]. In addition, the position of our FS for a finite Γ_0 around $\mathbf{k} = (0.8\pi, 0)$ slightly shifts to a smaller momentum in Supplementary Fig. 5, because of the shift of the chemical

potential due to the presence of $\Gamma_{\mathbf{k}}$. Although this shift comes from the charge conservation and is a similar mechanism of the shift of the chemical potential with temperature, our obtained shift might be an artifact of the simplicity of the present analysis, which neglects incoherent contributions to the spectral function $A_{\pm}(\mathbf{k}, \omega)$ [see Supplementary Eq. (17)]. In this sense, the present analysis should be regarded as a demonstration of the asymmetry of the broadening in $n(\mathbf{k})$ around $\mathbf{k} = (\pi, 0)$ and of the enhancement of $n(\mathbf{k})$ there. These come from the underlying band structure near the Fermi energy and thus are expected to be robust features. While we have introduced the damping of quasiparticles $\Gamma_{\mathbf{k}}$ to capture the pseudogap phenomenology, a result similar to Supplementary Fig. 5 is also obtained by invoking a gap in the electronic dispersion when its energy scale is comparable to our $\Gamma_{\mathbf{k}}$.

We also present in Supplementary Fig. 6 the corresponding results for $\alpha = 0$, namely for the conventional FS reported by ARPES (Ref. 8). $n(\mathbf{k})$ crosses the FS at $\mathbf{k} = (\pi, 0.14\pi)$ and $(0.43\pi, 0.43\pi)$. The broadening due to the damping of quasiparticles is visible around $\mathbf{k} = (\pi, 0)$ and is pronounced along the $(0, 0)$-$(\pi, 0)$ direction more than the $(\pi, 0)$-(π, π) direction, because the velocity along the $(0, 0)$-$(\pi, 0)$ direction is smaller as we have explained in Supplementary Eqs. (19) and (20). Since $\mathbf{k} = (\pi, 0)$ is located inside the FS, $n(\mathbf{k})$ is suppressed there due to the damping of quasiparticles, in sharp contrast to the case in Supplementary Fig. 5.

Supplementary Note 8: Degree of nematicity

As seen in Supplementary Eqs. (9) and (10), nematicity is parameterized by α in our formalism. We have taken $\alpha = 0.36$ in Fig. 4 to be consistent with our data. The degree of the nematicity, however, cannot be determined uniquely from the present data because of a rather broad feature of $n(\mathbf{k})$ and its derivative. In fact, an equally good fit is obtained in $0.32 \lesssim \alpha \lesssim 0.40$ at 300 K as shown in Supplementary Fig. 7. The situation is also the same at 150 K and we may invoke a value of α at least in $0.30 < \alpha < 0.44$, as was demonstrated in Fig. 6b, c. The corresponding data of $|\nabla n(\mathbf{k})|$ is shown in Supplementary Fig. 8a, b.

Supplementary Note 9: Effect of k_z dispersion

In the actual Compton scattering measurements, the spectrum is integrated along the k_z direction [Supplementary Eq. (14)]. It is therefore insightful to clarify the effect of the

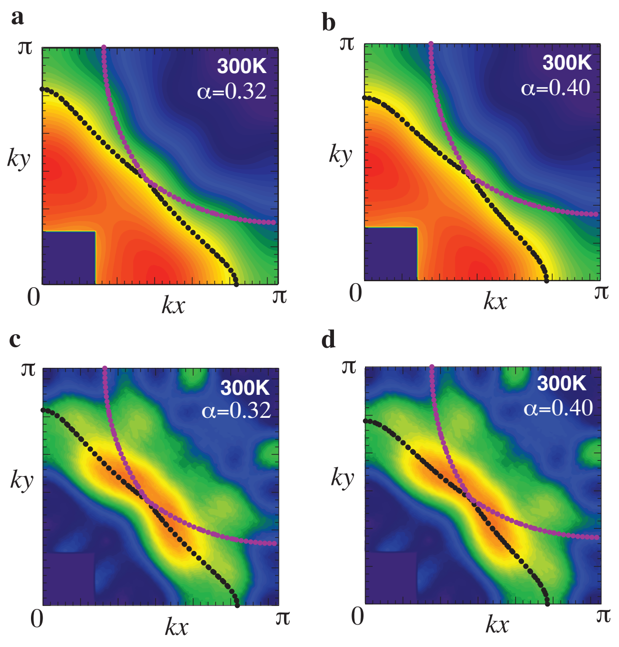

Supplemenatary Fig. 7. Interpretation of the observed momentum distribution function $n(\mathbf{k})$ at 300 K with a different degree of nematicity: $\alpha = 0.32$ (**a**) and 0.40 (**b**). **c** and **d** The corresponding data of the derivative of $n(\mathbf{k})$.

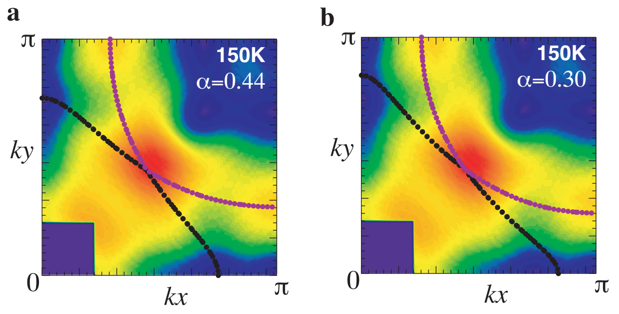

Supplemenatary Fig. 8. Interpretation of the observed maps of the derivative of momentum distribution function $n(\mathbf{k})$ at 150 K with a different degree of nematicity: $\alpha = 0.44$ (**a**) and 0.30 (**b**). The corresponding maps of $n(\mathbf{k})$ were shown in Fig. 6b, c.

k_z integration. Supplementary Fig. 9 compares $n(\mathbf{k})$ for $k_z = 0$ with that after integration with respect to k_z; the FSs for $k_z = 0$ are also superposed there. It is clear that the effect of k_z integration is very weak and $n(\mathbf{k})$ in Supplementary Fig. 9a is well captured in terms of the FSs for $k_z = 0$ even if we take $\tilde{t}_z$ larger than the realistic value.

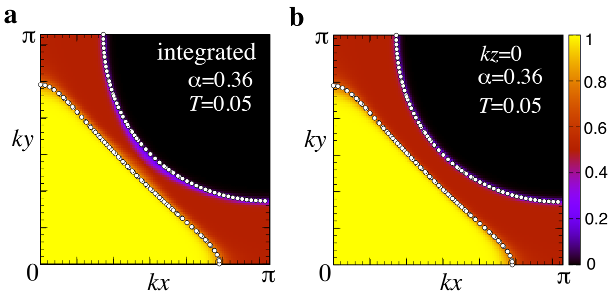

Supplemenatary Fig. 9. Momentum distribution function $n(\mathbf{k})$ of the Hamiltonian (3) for nematicity $\alpha = 0.36$ and interlayer hopping $\tilde{t}_z = 0.1$ at temperature $T = 0.05$ and doping $\delta = 0.08$: (**a**) k_z dependence is integrated [see Supplementary Eq. (14)] and (**b**) $k_z = 0$ is taken. The Fermi surfaces obtained for $k_z = 0$ are superposed on both figures.

Supplementary Note 10: Magnitude of *c*-axis dispersion

As discussed in the Supplementary Note 2, the actual value of $\tilde{t}_z$ is not known for LSCO with $x = 0.08$. As long as $\tilde{t}_z$ is sufficiently small, a difference between the *c*-axis dispersions [Supplementary Eqs. (11) and (12)] is minor. On the basis of a comparison between our proposed FS (Fig. 3) and the observed momentum distribution function presented in the main text, we may estimate $\tilde{t}_z \lesssim 0.01$ at 300 K. On the other hand, at 150 K it is possible to invoke a relatively large value such as $\tilde{t}_z \approx 0.05$ for the *c*-axis dispersion Supplementary Eq. (11). The resulting FSs for $k_z = 0$ are superposed on Supplementary Fig. 10. Compared with Fig. 4b, d, where we took $\tilde{t}_z = 0$, the major difference appears around $\mathbf{k} = (0.45\pi, 0.45\pi)$

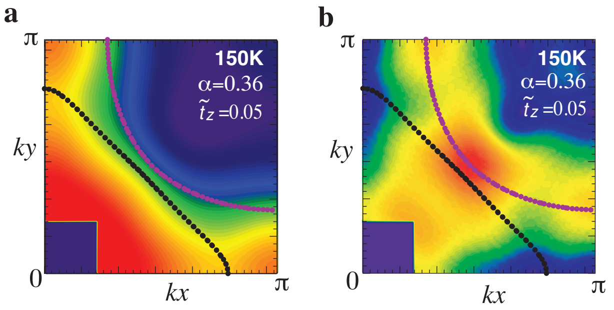

Supplemenatary Fig. 10. Interpretation of the observed maps of the momentum distribution function $n(\mathbf{k})$ (**a**) and its derivative $|\nabla n(\mathbf{k})|$ (**b**) at 150 K in terms of the Fermi surfaces with nematicity $\alpha = 0.36$ and $k_z = 0$ for $\tilde{t}_z = 0.05$ in Supplementary Eq. (11); the corresponding Fermi surface for $\tilde{t}_z = 0$ is shown in Fig. 4b, d.

due to the sizable interlayer coupling. Still the outer FS almost perfectly agrees with our data and in addition, the $\mathbf{k}_F$ dependence of $n(\mathbf{k}_F)$ is also weak entirely along the inner FS. We can conclude that the FSs with $\tilde{t}_z = 0.05$ are also consistent with our data. While the c-axis dispersion Supplementary Eq. (12) with $c_0 = 0$ vanishes around $\mathbf{k} = (0.45\pi, 0.45\pi)$ because of the factor of $(\cos k_x - \cos k_y)^2$, a split between the outer and inner FSs there is easily obtained by considering disorder effects which disrupt the symmetry of the bonding orbital[14]. In this case, one may invoke a finite value of c_0 in Supplementary Eq. (12).

Supplementary Note 11: Compton scattering for $x = 0.15$ and 0.30

We have focused on the doping rate $x = 0.08$ so far and showed that the FS can be strongly deformed by the underlying nematicity, but the bulk FSs recover the fourfold symmetry. It is natural to ask how the FS deformation evolves with increasing doping. At a fixed temperature, nematic correlations are expected to be less pronounced with increasing doping and eventually a conventional FS suggested by ARPES may be realized in heavily overdoped LSCO such as $x = 0.30$. This tendency also collaborates on the Z-point phonon mode, which is present in $x \leq 0.21$ (Refs. 6 and 15).

In this Note 11, we shall present data that the nematicity indeed becomes smaller at $x = 0.15$ and almost vanishes at $x = 0.30$ by performing additional Compton scattering measurements. To make a consistent comparison with the data at $x = 0.08$, we choose a temperature 300 K instead of 150 K. This is because a signature of short-range charge order is reported above the pseudogap temperature at $x = 0.15$ (Ref. 16) and we can safely avoid potential complications from that.

Supplementary Fig. 11a, b shows maps of $n(\mathbf{k})$ at $x = 0.15$ and 0.30 measured by our Compton scattering. The obtained map at $x = 0.15$ is similar to that for $x = 0.08$ at 300 K (Fig. 1a), implying that the underlying FSs are also similar to each other. Note that the FS at $x = 0.15$ is indeed very similar to that at $x = 0.08$ in the conventional picture, too[17]. In contrast, the map at $x = 0.30$ is different from those for $x = 0.08$ and 0.15 at 300 K, suggesting different shapes of FSs at $x = 0.30$. In fact, the conventional picture[17] tells that the electron-like FS is realized at $x = 0.30$ and the hole-like FS is at $x = 0.08$ and 0.15.

Under the condition of $\alpha = \alpha'' = 0$, $\tilde{t}''/\tilde{t}' = -1/2$, and $\tilde{t}_z = 0$ [see Supplementary Eqs. (9)-(12)], we first determine the actual band parameters $\tilde{t}'$ and $\tilde{t}''$ to reproduce the FS

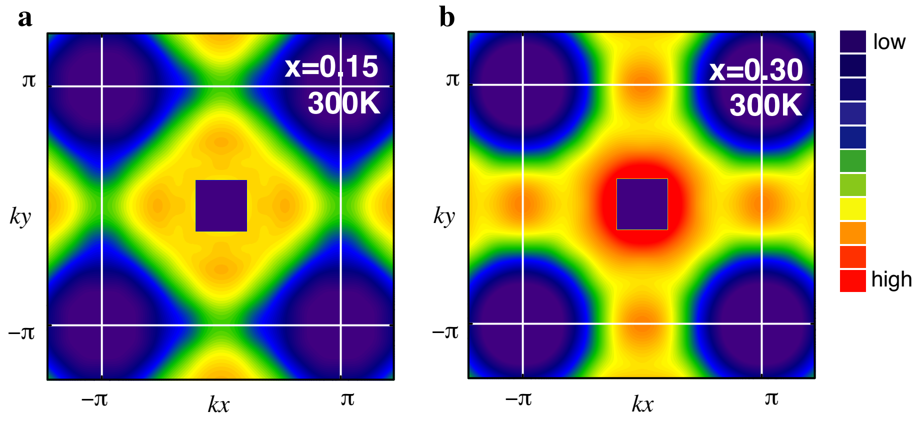

Supplemenatary Fig. 11. Images of the momentum distribution function $n(\mathbf{k})$ by high-resolution Compton scattering. Maps of $n(\mathbf{k})$ in the first Brillouin zone at 300 K for $La_{2-x}Sr_xCuO_4$ with $x = 0.15$ (**a**) and 0.30 (**b**). The results for $x = 0.08$ are shown in Fig. 1. The color scale represents the relative intensity.

proposed by ARPES[8,17]. We obtain

$$\tilde{t}' = -0.12\tilde{t}, \quad \tilde{t}'' = 0.06\tilde{t} \tag{21}$$

for both $x = 0.15$ and 0.30. The band parameters are different from those at $x = 0.07$ [see Supplementary Eq. (13)]. This doping dependence is well known in the tight-binding fit to ARPES data in La-based cuprates[17]. While we shall also consider various c-axis dispersions [Supplementary Eqs. (11) and (12)] to perform a precise analysis as much as possible, the essential feature of our Compton scattering data is captured already without considering the c-axis dispersion.

Analysis of $n(\mathbf{k})$ for $x = 0.15$

In Supplementary Fig. 12a, we superpose the FS proposed by ARPES[8]. Along the FS, $n(\mathbf{k}_F)$ is almost constant in an extended region around $\mathbf{k} = (0.43\pi, 0.43\pi)$. This nice agreement between ARPES and our Compton data is, however, broken around $\mathbf{k} = (\pi, 0)$ and $(0, \pi)$. This unsatisfactory aspect is resolved by considering FS deformation from the nematicity as shown in Supplementary Fig. 12b. The inner FS is fully consistent with the map of $n(\mathbf{k})$. The outer FS is also almost consistent with our data, although a small region around $\mathbf{k} = (0.43\pi, 0.43\pi)$ may not be so perfect. The agreement is improved when we introduce the c-axis dispersion in Supplementary Eq. (11) as shown in Supplementary

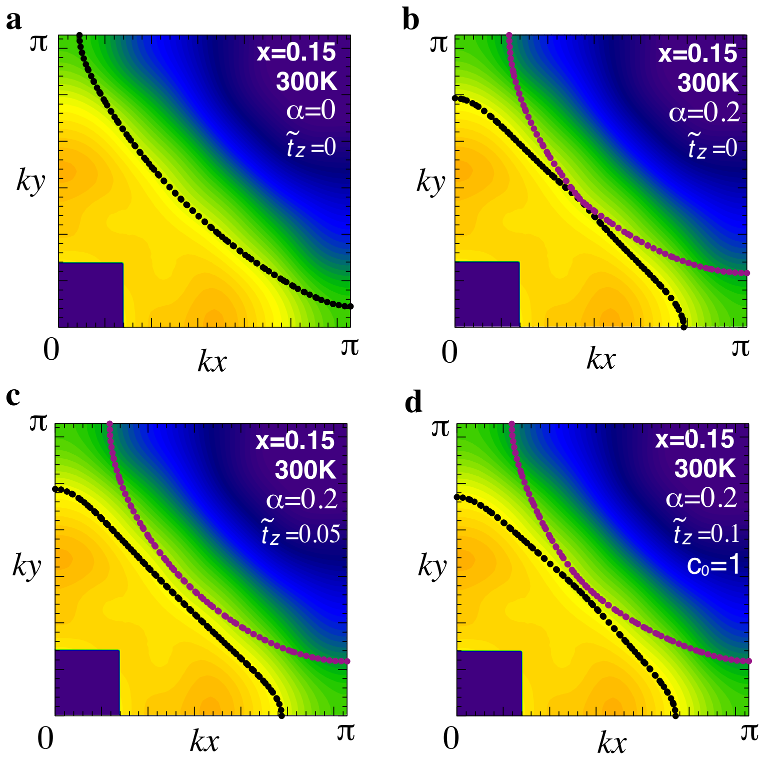

Supplemenatary Fig. 12. Interpretation of the observed maps of the momentum distribution function $n(\mathbf{k})$ for $x = 0.15$ at 300 K. Expected Fermi surfaces are superposed on the maps. **a** Fermi surface (FS) proposed by angle-resolved photoemission spectroscopy[8]. **b-d** FSs with $\alpha = 0.2$ in the nematic scenario. The c-axis dispersion is neglected in (**b**), and Supplementary Eqs. (11) and (12) for $k_z = 0$ are considered in (**c**) and (**d**), respectively.

Fig. 12c. The values of α and $\tilde{t}_z$ cannot be determined uniquely and a reasonably good agreement is achieved in $0.16 \lesssim \alpha \lesssim 0.25$ and $0 \lesssim \tilde{t}_z \lesssim 0.05$. Actually, a larger $\tilde{t}_z$ would be more consistent with our data, but the splitting of the hole- and electron-like FSs around $\mathbf{k} = (0.43\pi, 0.43\pi)$ becomes sizable, which does not seem to be supported by ARPES data[8]. A choice of a different c-axis dispersion [Supplementary Eq. (12)] yields essentially the same results (Supplementary Fig. 12d) even if we introduce $c_0 = 1$ in Supplementary Eq. (12) as long as $0 \lesssim \tilde{t}_z \lesssim 0.1$. Compared to the case at $x = 0.08$, a value of α becomes smaller at $x = 0.15$. This smaller α comes from weaker nematic correlations and weaker lattice anisotropy with carrier doping.

Analysis of $n(\mathbf{k})$ for $x = 0.30$

The FS proposed by ARPES[17] is superposed on our map of $n(\mathbf{k})$ in Supplementary Fig. 13a. Along the FS, $n(\mathbf{k}_F)$ is almost constant, although the agreement seems less satisfactory around a region $\mathbf{k} = (0.15\pi, 0.7\pi)$ and $(0.7\pi, 0.15\pi)$. To consider whether this is reasonably acceptable, we estimate the value of $n(\mathbf{k})$ along the FS shown in Supplementary Fig. 13a by assuming that $n(\mathbf{k}_F) = 0.5$ at $\mathbf{k}_F = (0.4\pi, 0.4\pi)$. The result is shown in Supplementary Fig. 13b. It turns out that $n(\mathbf{k}_F)$ varies very sightly around 0.5 along the expected FS, which indicates that the FS proposed by ARPES[17] agrees with our Compton scattering data. This conclusion is reasonable because the spectral function $A(\mathbf{k}, \omega)$ is rather sharp in ARPES at $x = 0.30$ (Ref. 17) and thus there is not much room to invoke additional physics that ARPES potentially misses. In addition, our conclusion is consistent with the previous

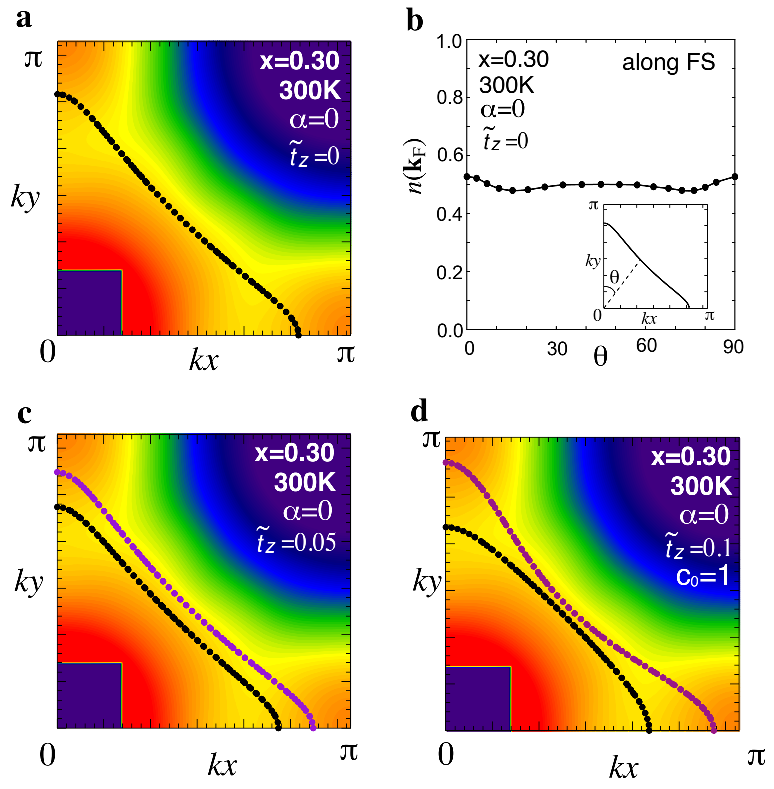

Supplemenatary Fig. 13. Interpretation of the observed maps of the momentum distribution function $n(\mathbf{k})$ for $x = 0.30$ at 300 K. Expected Fermi surfaces are superposed on the maps. **a** Fermi surface (FS) proposed by angle-resolved photoemission spectroscopy[17]. **b** $n(\mathbf{k}_F)$ along the expected FS shown in (**a**). The value of $n(\mathbf{k}_F)$ is normalized to be 0.5 at $\mathbf{k}_F = (0.4\pi, 0.4\pi)$ and the angle θ is defined in the inset. **c** and **d** FSs in the presence of the c-axis dispersions Supplementary Eqs. (11) and (12), respectively, for $k_z = 0$.

Compton scattering for $x = 0.30$ (Ref. 18). Inclusion of the c-axis dispersion such as Supplementary Eqs. (11) and (12) does not alter our conclusion as seen in Supplementary Fig. 13c, d. Note that in general there should exist two FSs at a given k_z in LSCO because the unit cell contains two CuO_2 planes reflecting the body-centered tetragonal crystal structure. If we invoke a larger $\tilde{t}_z$, a value of $n(\mathbf{k})$ tends to vary along the outer FS especially around $\mathbf{k} = (\pi, 0)$ and $(0, \pi)$. The reason why the momentum distribution function $n(\mathbf{k})$ is enhanced around $\mathbf{k} = (\pi, 0)$ and $(0, \pi)$ is left to further studies.

While it seems unlikely to invoke the nematic physics at $x = 0.30$, it is worth checking whether our data are indeed consistent with this expectation. We found that essentially the same FSs are obtained as those in Supplementary Fig. 13c, d for the same band parameters except for a finite value of $\alpha \lesssim 0.05$. In this sense, our data cannot exclude possible nematicity also at $x = 0.30$, but with a small α if there is.

---

[1] Here $\mathbf{k} > \mathbf{k}_F$ and $\mathbf{k} < \mathbf{k}_F$ should be understood as the momentum $\mathbf{k}$ outside and inside the Fermi surface, respectively.

[2] Stephan, W. & Horsch, P. Fermi surface and dynamics of the t-J model at moderate doping. *Phys. Rev. Lett.* **66**, 2258–2261 (1991).

[3] Pruschke, T. & Shiba, H. Correlation functions and critical exponents in the one-dimensional anisotropic t-J model. *Phys. Rev. B* **44**, 205–216 (1991).

[4] Sato, R. & Yokoyama, H. Band-renormalization effect on superconductivity and antiferromagnetism in two-dimensional t-J model. *J. Phys. Soc. Jpn.* **87**, 114003 (2018).

[5] Radaelli, P. G. *et al.* Structural and superconducting properties of $La_{2-x}Sr_xCuO_4$ as a function of Sr content. *Phys. Rev. B* **49**, 4163–4175 (1994).

[6] Kimura, H., Hirota, K., Lee, C.-H., Yamada, K. & Shirane, G. Structural instability associated with the tilting of CuO_6 octahedra in $La_{2-x}Sr_xCuO_4$. *J. Phys. Soc. Jpn.* **69**, 851–857 (2000).

[7] Horio, M. *et al.* Three-dimensional fermi surface of overdoped La-based cuprates. *Phys. Rev. Lett.* **121**, 077004 (2018).

[8] Yoshida, T., Hashimoto, M., M. Vishik, I., Shen, Z.-X. & Fujimori, A. Pseudogap, superconducting gap, and fermi arc in high-T_c cuprates revealed by angle-resolved photoemission spectroscopy. *J. Phys. Soc. Jpn.* **81**, 011006 (2012).

[9] Andersen, O., Liechtenstein, A., Jepsen, O. & Paulsen, F. LDA energy bands, low-energy hamiltonians, t', t'', $t_\perp(k)$, and $J_\perp$. *J. Phys. Chem. Solids* **56**, 1573–1591 (1995).

[10] Ito, T., Takagi, H., Ishibashi, S., Ido, T. & Uchida, S. Normal-state conductivity between CuO_2 planes in copper oxide superconductors. *Nature* **350**, 596–598 (1991).

[11] Yamase, H. & Kohno, H. Magnetic excitation of t-J model with quasi-one-dimensional fermi surface - possible relevance to LSCO systems. *J. Phys. Soc. Jpn.* **70**, 2733–2745 (2001).

[12] Timusk, T. & Statt, B. The pseudogap in high-temperature superconductors: an experimental survey. *Reports on Progress in Physics* **62**, 61–122 (1999).

[13] Hashimoto, M. *et al.* Distinct doping dependences of the pseudogap and superconducting gap of $La_{2-x}Sr_xCuO_4$ cuprate superconductors. *Phys. Rev. B* **75**, 140503(R) (2007).

[14] Xiang, T. & Wheatley, J. M. c axis superfluid response of copper oxide superconductors. *Phys. Rev. Lett.* **77**, 4632–4635 (1996).

[15] Birgeneau, R. J. *et al.* Antiferromagnetic spin correlations in insulating, metallic, and superconducting $La_{2-x}Sr_xCuO_4$. *Phys. Rev. B* **38**, 6614–6623 (1988).

[16] Wen, J. J. *et al.* Observation of two types of charge-density-wave orders in superconducting $La_{2-x}Sr_xCuO_4$. *Nature Communications* **10**, 3269 (2019).

[17] Yoshida, T. *et al.* Systematic doping evolution of the underlying fermi surface of $La_{2-x}Sr_xCuO_4$. *Phys. Rev. B* **74**, 224510 (2006).

[18] Al-Sawai, W. *et al.* Bulk fermi surface and momentum density in heavily doped $La_{2-x}Sr_xCuO_4$ using high-resolution compton scattering and positron annihilation spectroscopies. *Phys. Rev. B* **85**, 115109 (2012).
