# Supplementary material for: Fermi surface in La-based cuprate superconductors from Compton scattering imaging
Source: Nat Commun. 2021 Apr 13;12:2223. doi: 10.1038/s41467-021-22229-6 (PMC8044246; doi:10.1038/s41467-021-22229-6)
Supplement: Supplementary file 2 — Peer Review File [file 41467_2021_22229_MOESM2_ESM.pdf]

REVIEWER COMMENTS

Reviewer #1 (Remarks to the Author):

This paper reports Compton scattering data on the Fermi surface and momentum distribution function of the strongly underdoped high-temperature superconductor La(2-x)Sr(x)CuO4. Maps of the Compton profiles were measured at two temperatures (150 and 300 K) and were found to be surprisingly different. Moreover, the nature of the difference is different from what one would expect based on the opening of the momentum-dependent “pseudogap” revealed in previous work. The authors have performed detailed model calculations to explain this observation. The scenario they come up with is based on an intricate interplay of three different factors: the pseudogap, nematic fluctuations of the Fermi surface, and coupling to a low-energy phonon mode. This is certainly a clever and intriguing explanation, but the model is severely underconstrained by the two data sets included in the paper. A minimal data set required to firmly support or refute key aspects of the model would have to include data deep in the pseudogap phase, as well as data in the overdoped regime where both the pseudogap and the nematic fluctuations are believed to be absent. (Note that Ref. 10 does report Compton scattering data on overdoped La(2-x)Sr(x)CuO4, but only at room temperature and only along a single direction in momentum space. This data set is too sparse to be useful for the comparison required here.)

For these reasons, I cannot recommend publication of the paper in its current state. I recommend reconsideration, if the authors include a more complete set of data that allows a meaningful comparison with predictions derived from the model.

Reviewer #2 (Remarks to the Author):

Compton scattering is a bulk technique which is capable of revealing the electron momentum distribution and the Fermi surface of metallic systems. The current Compton study on underdoped LSCO shows that there is a change in the occupation number within the Brillouin zone at temperatures above and below 200K, the temperature associcated with the appearance of the pseudogap. At both temperatures, the observed momentum densities cannot straightforwardly be explained in terms of the conventional picture of just a (pi,pi)-centered hole Fermi surface, and additionally, there is extra smearing which
appears in the 150K measurement (particularly clear in the derivative plots of Fig.2). Using a description of the Fermi surface deformed differently by nematicity in alternate planes (such that the Fermi surface retains fourfold symmetry), they argue that such a model explains their Compton scattering data.

Furthermore, the authors point out that they don't consider that their results are incompatible with the ARPES results, and suggest that features in those spectra are consistent with their picture of two Fermi surfaces due to the nematicity.

This is new, and likely to be of significant interest to a wide community. More broadly, it showcases the power and complementary nature of the Compton scattering technique (to quantum oscillations and ARPES) which could be used to provide new insights
into other strongly correlated materials. Their work is clearly presented, and their arguments well formed and compelling. The data and analysis procedures are thorough.

I therefore recommend that the manuscript be accepted in Nature Communications. Some specific

comments are outlined below.

1. The impact of a gap (CDW, SDW, superconducting, pseudogap etc.) on the momentum distribution has been considered before, and for completeness I would like to draw the authors' attention to the following two papers :
B L Gyorffy et al. J. Phys.: Condens. Matter 1 SA119 (1989) (doi: 10.1088/0953-8984/1/SA/016)
J Friedel and M Peter EPL 8 79 (1989) (doi: 10.1209/0295-5075/8/1/014)

2. The notation should be consistent. In most places the momentum distribution function is denoted as n(k), but n_k is also used in the introduction.

3. In Section II (Results), there is a discussion about the momentum distribution function being contributed to by all bands, but that the Fermi surface influences the k dependence. There should be some reference to how n(k) (in crystal momentum space) is obtained from the measured Compton profiles (which represent real momentum, not crystal momentum) ; this is the so-called Lock-Crisp-West theorem. While this theorem is exact for non-interacting electrons, it is an approximation for interacting electrons. This could be in the Supplementary Information.

4. In Fig.1 and some subsequent figures, the region close to the center of the Brillouin zone is excluded. The authors say that this region has a large experimental error. They should explain that this is due to the direct Fourier scheme that they have employed to tomographically reconstruct which produces large unphysical oscillations at low momentum. Also, in the caption to Fig. 1, it says that (c) is a difference
between two temperatures. It must be "150K - 300K", but this should be made explicit.

5. Line 157 : In the discussion of scenario A, there is mention of the impact of the pseudogap on n(k) at (pi,0) and (0,pi) which are described as being "inside the conventional Fermi surface". I'm a little confused by this phrasing as I thought that the conventional hole-like Fermi surface was centered at (pi,pi) and thus (pi,0) and (0,pi) are outside the (hole) Fermi surface i.e. in the occupied region of the Brillouin zone. It is therefore ok to talk about n(k) being suppressed in this region by the smearing due to the pseudogap, but the description "inside the conventional Fermi surface" is misleading.

6. Line 306 : It says 3 x 105 (one hundred and five) counts were in the Compton peak. I presume that this means 3 x 10^5, which implies that this is high statistics data.

****
Response to Reviewer #1
****

Thank you very much for reviewing our manuscript and providing us a constructive comment: “I recommend reconsideration, if the authors include a more complete set of data that allows a meaningful comparison with predictions derived from the model.”.

As a function of doping at a fixed temperature, nematic correlations are expected to be less pronounced with increasing doping and thus a conventional two-dimensional electron-like FS may be realized eventually in heavily overdoped LSCO. This also collaborates on the Z-point phonon mode, which is present in $x < 0.21$. In this case, our model predicts that FS deformation is weakened with increasing doping, that is, our parameter alpha becomes smaller. Therefore we present additional Compton scattering data at x=0.15 and 0.30 at room temperature; a choice of lower temperature could produce additional complications due to charge and spin ordering tendencies in LSCO, which are beyond the scope of the present work (please also see line 83 - 92 in the main text). While details are given in Supplementary Material K, the essential points are the following.

The data at x=0.15 turn out to be similar to those at x=0.08, suggesting similar underlying FSs at both doping rates. In fact, we confirm that deformed FSs with weaker nematicity are compatible with our data.

The data at x=0.30 look different from those at x=0.08 and 0.15, suggesting the underlying FSs different from those at x=0.08 and 0.15. While we cannot exclude possibly tiny deformation of the FS, our data are compatible with the conventional electron-like FS, which agrees with the FS obtained by ARPES as well as the implication obtained previously in Compton scattering (Ref. 12).

On the other hand, as a function of temperature at a fixed doping, we have not obtained insights into how the nematicity should evolve as we discussed in the context of Fig. 6. Needless to say, it should be exciting to study more the temperature dependence of n(k) since we may obtain valuable insights into i) the interplay of the pseudogap and nematicity, ii) a role of spin-charge stripes, and iii) a possible interplay of nematicity and charge stripes, which are, however, separate issues from the present work. The present work is the first Compton scattering study to explore the underlaying FS in the underdoped LSCO. We think that our results are already worth reporting and will stimulate many researchers who are interested in cuprates, nematicity, and a complementary nature of Compton scattering to ARPES and quantum oscillations. Please also see our concluding remark session.

The reviewer reminds us of the importance to clarify the validity of our model (Fig.3) and its limitation. Hence we have mentioned them in the concluding remark section.

We hope that the validity of our model is reinforced further by the additional data at x=0.15 and 0.30 and that the reviewer 1 also finds our arguments and analyses compelling and thorough.

****

Response to Reviewer #2

****

Thank you very much for reviewing our manuscript and providing us valuable and thoughtful comments. We also appreciate his/her insight into the significance of the present work: “This is new, and likely to be of significant interest to a wide community. More broadly, it showcases the power and complementary nature of the Compton scattering technique (to quantum oscillations and ARPES) which could be used to provide new insights into other strongly correlated materials.”

1.
We have cited the two references in the introduction (line 66).

2.
We now use the same notation n(k) as the momentum distribution function (line 62 and 64).

3.
Following the reviewer’s comment, we have explained the LCW theorem in the first paragraph in Results section (line 96) and the second paragraph in Experimental method section (line 351-353).

4.
Following the reviewer’s comment, we have explained the errors of experimental n(k) in the second paragraph in Experimental method section (line 349-351).

We have mentioned “150 K - 300 K” in Fig.1 (c)

5.
line 157 (now 164):
are located inside the conventional FS
—>
are occupied by electrons for the conventional FS

6.
As the referee mentions, 3 x 10^5 is correct (line 344).

Reviewers' comments:

Reviewer #1 (Remarks to the Author):

I had recommended reconsideration of the manuscript, if the authors were able to present additional Compton scattering data at higher doping levels, where the electronic structure is known to be amenable to a more conventional description than in the strongly underdoped regime for x = 0.08. In line with this recommendation, the authors have included two additional momentum-distribution maps for doping levels x = 0.15 and 0.30. Unfortunately, these data sets do not provide the kind of reassurance I was expecting. Oddly, the data for 0.15 look almost identical to those at 0.08 at room temperature, even though the doping level is almost a factor of two higher. Even more strangely, the room-temperature data for x = 0.30 look very similar to those for x = 0.08 at 150 K. The authors are somehow able to fit all of these data, and the similarities pointed out above are (implicitly) explained as pure coincidences. However, given the sparsity of the data and the multitude of parameters, these fits do not inspire a lot of confidence, so that the manuscript at hand falls short of convincing the reader of the power of Compton scattering as a probe of electronic correlations in cuprates and other complex materials.

To clear this bar, temperature dependent data for x = 0.15 and 0.30 and/or lower-temperature data for x = 0.08 would be extremely helpful. The authors' justification for not including such data in their rebuttal letter is not persuasive. They say that "a choice of lower temperature could produce additional complications due to charge and spin ordering tendencies in LSCO". However, charge and spin order do not actually occur for optimally doped and overdoped LSCO, whereas they do in fact occur for x = 0.08.

In addition, the room-temperature data should be presented and analyzed in a more transparent fashion. In the present version, it is difficult to find out which hopping parameters were actually used to calculate the theoretical plots for x = 0.08, but if the parameters of Eq. 13 were used, they are actually different from those used for the higher doping levels (Eq. 21). It would be interesting to present doping-differential measurements in a manner similar to Fig. 1c, along with calculations based on a minimum of assumptions (i.e. fixed band structure and variable band filling). Further assumptions (including doping-dependent nematicity and/or c-axis hopping) can then be added systematically.

Reviewer #2 (Remarks to the Author):

The revised manuscript (and substantial additions to the Supplementary information related to the additional data) is significantly improved. The new data bolsters the interpretation developed on the basis of the 0.08 doping. My previous comments have been satisfactorily addressed.

****
Response to Reviewer #1
****
We appreciate very careful reading of the manuscript including Supplementary Material. It is quite regrettable that the reviewer #1 was not satisfied with our response in spite of our effort to collect the additional Compton scattering data and to perform detailed analyses.

First of all, we would like to highlight five crucial points in the present communications with the reviewer #1. i) Our aim is not to convey the power of Compton scattering measurements from a general perspective. ii) The present work is the first measurement to reveal the FS shape in the underdoped cuprates and we successfully detect for the first time a signature of the "missing part" of the FS, which has not been detected by ARPES so far. This is the core part of the present work. iii) In addition, we found that the obtained data can be interpreted consistently in terms of nematicity. Interestingly, in contrast to Y-based cuprates, the resulting electronic property does not exhibit anisotropy in the La-based system. iv) Our data indeed reproduce the conventional picture/understanding when nematicity is not present. First, our Compton scattering data reproduce very well the FS proposed by ARPES near (pi/2, pi/2), where the effect of nematicity is weak even in the underdoped region. Second, nematicity is expected to become weaker with increasing doping as the reviewer #1 also expects. Our data at x=0.15 and 0.30 indeed show that our maps become more consistent with the conventional FS with increasing the doping. v) The tight-binding dispersion is obtained by fitting to existing ARPES data where no nematicity was considered. Our free parameter is only one, namely nematicity, which is described by alpha in the present work and is obtained by fitting to our Compton scattering data.

1. I had recommended reconsideration of the manuscript, if the authors were able to present additional Compton scattering data at higher doping levels, where the electronic structure is known to be amenable to a more conventional description than in the strongly underdoped regime for x = 0.08. In line with this recommendation, the authors have included two additional momentum-distribution maps for doping levels x = 0.15 and 0.30. Unfortunately, these data sets do not provide the kind of reassurance I was expecting.

Our data indeed reproduce the conventional picture/understanding when nematicity is very weak, which can be understood even without studying a different doping rate. Nematicity is related to xy-symmetry breaking and thus characterized by d_{x^2-y^2} symmetry. In this case, the effect of nematicity vanishes along the diagonal direction in momentum space, namely along the axis ky = \pm kx. Hence one expects that our data should reproduce a conventional description there, which means that our data should be consistent with the ARPES data around (pi/2,pi/2). Fig. 2(a)(b), Fig. 18(a), and Fig.19(a) clearly show that our Compton scattering data reproduce very well the FS proposed by ARPES in a region around (pi/2, pi/2). Given that ARPES spectra are very sharp and very clear near (pi/2, pi/2), this is hard evidence to secure our data.

Our data indeed perfectly match with what one expects from the doping dependence. The data at x=0.15 is similar to that at x=0.08 at T=300 K. This is the very expectation of many researchers, because the FS proposed by APRES is very similar for both x=0.08 and 0.15. The data at x=0.30 is similar to that at x=0.08 at T=150 K. This is again the very expectation of many researchers, because roughly speaking, the electron occupation near "(pi,0)" and "(0,pi)" is reduced in both cases in different mechanisms: In the former case, the non-interacting FS becomes electronlike, whereas in the later case, the non-interacting FS is holelike and the pseudogap opens around (pi,0) and (0,pi) below T=200 K. If the precise statements are favorable, we need to consider additional effects: the quasi-particle damping or the gap formation on the nematic FSs at x=0.08 and thermal broadening effect for the electronlike FS at x=0.30. See Sec. II B 2 and Supplementary Material K 2.

In addition, we took data twice at each doping and temperature, and confirmed that the data are indeed reproducible. We also made our analysis rather critically and checked that our data and analyses are indeed consistent with the existing knowledge and reproduced the conventional picture obtained by ARPES when nematicity is expected to be weak. We also checked the agreement with the former Compton scattering data at x=0.30 (Ref. 12). We believe that these critical and careful analyses are easily read into our manuscript.

2. Oddly, the data for 0.15 look almost identical to those at 0.08 at room temperature, even though the doping level is almost a factor of two higher.

As mentioned in the point 1, the data at x=0.15 is not odd at all. Rather it perfectly agrees with one's expectation.

Because of the Luttinger theorem, the difference of the FS area between x=0.15 and 0.08 is 1-0.15: 1-0.08 = 0.85: 0.92 ~ 1: 1.08. Hence as a report in ARPES (see Fig.2(a)(b) and 18(a), and also Fig. 5(f) in Ref. 44), both FSs become similar to each other; a small difference appears only near (pi,0) and (0,pi), Hence the momentum distribution function also does. It is misleading to say "even though the doping level is almost a factor of two higher".

3. Even more strangely, the room-temperature data for x = 0.30 look very similar to those for x = 0.08 at 150 K.

As mentioned in the point 1, the data at x=0.30 is not strange at all. Rather it perfectly agrees with one's expectation.

4. The authors are somehow able to fit all of these data, and the similarities pointed out above are (implicitly) explained as pure coincidences.

They are not "pure coincidences" as mentioned in the point 1.

5. However, given the sparsity of the data and the multitude of parameters, these fits do not inspire a lot of confidence, so that the manuscript at hand falls short of convincing the reader of the power of Compton scattering as a probe of electronic correlations in cuprates and other complex materials.

Our data are not sparse at all. We emphasize that our study provides maps of the momentum distribution function in the 2D BZ for the first time in cuprates. Now we have four! data of such maps. Compared with the former Compton scattering data (Ref. 12), we have achieved now to obtain much more comprehensive, detailed, and fine data. Furthermore, we have elucidated what other probes such as ARPES missed.

We do not have multitude parameters. Practically the fitting parameter is just alpha only, namely the degree of nematicity; the other parameters are determined by the ARPES data.

Since the nematic scenario is naturally related to the interlayer coupling, too, we also considered the c-axis dispersion, which is, however, not known much in the literature. Hence we fitted it to our data as an endeavor to extract some new/ useful insight from our data. However, this is not relevant to the conclusions in the manuscript. In fact, the effect of the c-axis dispersion is neglected in the main text and discussed only in Supplementary Material as details. Hence we have revised Supplementary Material to emphasize more that the c-axis dispersion is not relevant to our major conclusions.

As we have mentioned in the point 1, the additional data are fully consistent with each other. The present Compton scattering data almost perfectly reproduce ARPES data around (pi/2, pi/2) and furthermore capture for the first time FS deformation by the underlying nematicity, which was not captured by ARPES. In this sense, we believe that Compton scattering is powerful. Nevertheless, we emphasize that it is not our aim to convince the readers of the power of Compton scattering. Such a general aim is beyond the scope of the present work. Instead, our messages are threefold as summarized in the first four paragraphs in Section III concluding remarks, which should be very valuable to the community.

6. To clear this bar, temperature dependent data for x = 0.15 and 0.30 and/or lower-temperature data for x = 0.08 would be extremely helpful. The authors' justification for not inclusding such data in their rebuttal letter is not persuasive.

We disagree with the reviewer #1. The aim of our work is to present a new aspect of the FS in cuprates that other probes have not revealed yet. The reviewer #1 seems to like to know whether Compton scattering itself really reproduces the well established phenomena and proposes "to clear this bar". However, it is already done. Please recall that the FS around (pi/2, pi/2) is believed to be rather conventional even in the underdoped region. We already showed that Compton scattering reproduces very well the FS around (pi/2, pi/2) revealed by ARPES at x=0.08 and now also at x=0.15, and in addition a whole FS at x=0.30. This is hard experimental evidence to secure our data.

It is unexpected also for us that we did not obtain the textbook-like momentum distribution function at x=0.30 where many people assume that the weak coupling theory is applicable. However, as we mentioned in Supplementary Material K, it is gradually recognized that such a naive assumption seems not correct even in the overdoped region. A good example there is a non-BCS-like relation between the critical temperature of superconductivity and the superfluid density at zero temperature; see Bozovic et al., Nature 536, 309 (2016) (now Ref. 60). We think that our Compton scattering data at x=0.30 is indeed in harmony with this recent insight that the overdoped region is not so conventional. This kind of insight has not been obtained yet in ARPES. Hence we feel that the data at x=0.30 can also be an example to show powerfulness of Compton scattering, although we do not intend to insist on that because it is not our aim of the present work.

7.
They say that "a choice of lower temperature could produce additional complications due to charge and spin ordering tendencies in LSCO". However, charge and spin order do not actually occur for optimally doped and overdoped LSCO, whereas they do in fact occur for x = 0.08.

As we mentioned in the last paragraph of Introduction in the manuscript, our choice of x=0.08 is made very judiciously to avoid potential complications of a study on the underlying FS in the underdoped region. In addition, we also need to take into account that Compton scattering is a high-energy probe and thus dynamical effects can also be detected. Given that our work is the first Compton scattering study, those potential complications should be avoided in the beginning. This was the reason why we chose x=0.08 at relatively high T and also x=0.15 and 0.30 at T=300 K to

strengthen our analysis and interpretation.

The reviewer #1's statements are not correct for x=0.08 and 0.15.

At x=0.08, there are no charge order (Ref. 20); magnetic order is, however, reported below a few K (Ref. 22). Hence the temperature should be relatively high to safely avoid dynamical effects from the magnetic order.

At x=0.15, short-range charge order is reported below a certain characteristic temperature around 70 K (Wen et al., Nat. Commun. 10, 3269 (2019), which is cited as Ref. 59 now). Furthermore the authors found that such signal persists even above the pseudogap temperature. To avoid this safely, the temperature should be high enough.

Needless to say, it would be nice if we can also clarify the role of magnetic and charge orders as well as their dynamical effects on the FS by Compton scattering for lower temperatures. However, that is beyond the scope of the present study.

It is not our aim to demonstrate that Compton scattering is a powerful tool on general ground. Rather we are interested in observing something that other probes have not yet observed, namely in performing experiments to detect the underlying FS in the underdoped cuprates. Further Compton scattering data at x=0.30 below T=300 K do not contribute to our aim.

8.
In addition, the room-temperature data should be presented and analyzed in a more transparent fashion. In the present version, it is difficult to find out which hopping parameters were actually used to calculate the theoretical plots for x = 0.08, but if the parameters of Eq. 13 were used, they are actually different from those used for the higher doping levels (Eq. 21). It would be interesting to present doping-differential measurements in a manner similar to Fig. 1c, along with calculations based on a minimum of assumptions (i.e. fixed band structure and variable band filling). Further assumptions (including doping-dependent nematicity and/or c-axis hopping) can then be added systematically.

Our major points, namely the most important scientific messages are mentioned in the main text, not in Supplementary Material. The reviewer #1 mentions details in Supplementary Material.

Our description and a logical flow are simple and systematic. First, our data are compared with the FS proposed by ARPES. Second, nematicity is considered.

We can say the same thing from the view of fitting, too. First we determine the hopping integral from the ARPES data. It is well known that a conventional tight-binding fitting to ARPES data gives hopping integrals depending on doping; see Ref. 44. Hence they also does in the present work. These fitting analyses are standard. Second, we consider the parameter alpha to study the effect of deformation of the FS. This is a new feature of the present work to elucidate the effect of nematicity. This nematicity is determined by fitting to our Compton scattering data.

Since the nematic scenario necessarily involves the insight into the interlayer coupling as shown in Fig.3, we also analyzed the c-axis dispersion as an endeavor to extract some insight into that from the present Compton scattering data. This is however not important to the major conclusions of the present work. Hence we neglected the effect of the c-axis dispersion in the main text. Additional details about that are, therefore, provided in Supplementary Material after discussing a comparison with ARPES data and a role of nematicity.

We do not think it insightful to present the doping-differential data. When doping varies, two factors change: the original FS proposed by ARPES, which does not contain nematicity, and the degree of nematiciy. The doping-differential data are not easy enough to convey the underlying physics. With such data, our description would become much less transparent.

While we have provided detailed explanation on the additional data at x=0.15 and 0.30, we think that it is necessary as a stringent test of the robustness of our claims and also of reliability of our data and analyses, which is owed a lot to the reviewer #1. We made an additional effort to revise Supplementary Material.

Still if our additional data make the reviewer #1 unhappy in spite of our explanation and effort, we would suggest that the reviewer #1 focus on a region around (pi/2, pi/2) where the effect of nematicity is weak. Hence one expects that the momentum distribution function measured by Compton scattering should be consistent in that region with the conventional FS suggested by ARPES. This is beautifully the case in a region around (pi/2, pi/2) in Fig. 2 (a) and (b) at x=0.08, Fig. 18 (a) at x=0.15, and Fig. 19 (a) at x=0.30.

In summary, we appreciate the reviewer #1's very careful reading of the manuscript, especially Supplementary Material. We have made further improvements as much as possible by taking all the comments from the reviewer #1 into account as seen in a separate file.

REVIEWERS' COMMENTS

Reviewer #2 (Remarks to the Author):

It seems that inclusion of the new data has led to some confusion, particularly around what changes you would expect with doping and the Luttinger volume. I am firmly in agreement with the authors' responses regarding their rebuttal of reviewer#1's comments on those new data.

The debate with reviewer#1 has, however, unquestionably sharpened the arguments presented in the manuscript and supplementary information. I believe that the comparison with ARPES is important, not least as an independent verification of those results with a different technique. In my opinion, the results presented appear robust
and contrary to the comments of reviewer#1 the new data (the collection of which represents a huge effort) add to the confidence in their data and its interpretation. I still think that the extraction of the nematicity with the single parameter "alpha" is very neat. It should be remembered that each k-space map displayed here is derived f
rom a number of directional Compton profiles and I do not believe that the data are "sparse".

I also agree with the authors that doping-differential measurements would not add to the story.

Reviewer #3 (Remarks to the Author):

The authors have investigated the Fermi surface of lanthanum cuprates with three different Sr dopings by means of Compton scattering: first with a doping of x=0.08 at 300 and 150K (under doped region), then at the suggestion of Referee #1 additionally for x=0.15 and x=0.30 (overdosed), but in the latter cases only at 300K. Reconstructed and refolded data are shown, i.e. the occupation number density (here called "momentum distribution function" n(k)).
The main focus of the paper is as in the original version of the manuscript on the under doped regime x=0.08. The transition temperature TC is 20K and the transition to the pseudogap phase occurs at about 200K, i.e. between the two measurement temperatures.
While in previous ARPES results a single hole-like Fermi surface was observed for x=0.07, now the main result of the present work is that the authors interpret their Compton data as showing that the Fermi surface is highly distorted and bifurcated with an inner and outer Fermi surface as a result of electronic nematicity (Fig. 3). This interpretation of the data is supported by detailed tight-binding calculations. Observed differences between n(k) at 300 and 150K are explained by the interplay between nematicity and the formation of the pseudogap. For the higher dopings, the authors conclude from their data that nematicity weakens with increasing doping, so that at x=0.30 the "usual" electron-like Fermi surface is restored.
The manuscript was revised after the first round of reviewers, and the revised version was recommended for publication by one of the two reviewers (Referee #2), while Referee #1 remains sceptical. The present review represents a third round of reviews.

Own comment:
For x=0.08, taking into account the methodologically “spongy” quality of the data, the interpretation seems to me to be quite conclusive. One can clearly see a discrepancy with the earlier ARPES data (Fig. 2) and the detailed analysis in the context of electronic nematicity seems conclusive, although the agreement between theory and experiment (Fig. 4) requires some good will. However, the authors address all possible concerns in detail and with, in my view, convincing arguments. Also, the argumentation on the formation of the pseudogap below 200K and the resulting consequences for the

150K data seems conclusive.
The authors claim to detect no anisotropy in the bulk Fermi surface despite strong anisotropy of the Fermi surface in each CuO2 layer (because the unit cell contains two CuO2 layers and the associated parts of the Fermi surface are perpendicular to each other, see Fig. 3). However, the authors only measured Compton profiles in equidistant directions between the [100] and [110] directions, i.e., only along one-eighth of the Brillion zone, and apparently obtained n(k) from this by symmetrisation in the entire 1-st Brillouin zone. With this procedure, in my opinion, a possible xy-anistropy cannot be determined at all. However, this is only a minor aspect that does not affect the main message of the paper.
I still find the interpretation of the data for x=0.15 reasonably convincing, especially with respect to reduced nematicity (expressed by the anisotropy parameter), although the margin on the parameter is obviously quite large.
Concerning the data for x=0.30 I find the interpretation somewhat problematic. Here, according to general expectation, the Fermi surface should no longer be subject to nematicity, and pseudogap formation should also be absent, i.e., two essential aspects determining the Fermi surfaces at x=0.08 are missing. Nevertheless, the corresponding n(k) maps for x=0.08 at 150K as well as for x=0.30 at 300K look remarkably similar. This is noticed by both the authors and the referee. Nevertheless, the authors conclude that the underlying Fermi surfaces are fundamentally different and that the similarity of the occupation number densities arises as a result of different effects virtually by chance. For example, in Fig. 19a, the authors believe they can see a good match with earlier ARPES data, which again I do not find very convincing. Critically, I find that the authors first use two Supplementary sections (C and D) on it to prove that thermal broadening has no significant effect on the n(k) maps at 300K, at least at x=0.08 and x=0.015. On the other hand, the same thermal broadening is said to be responsible for relatively significant deviations of the n(k) map for x=0.30 and 300K from the expected picture. The authors provide an explanation for this in Supplement K and explain it with a dependence of the broadening effect on doping, however, this explanation seems to me somewhat constructed. But, again, the essential topic of the paper is the material at x=0.08, and the comparison with x=0.30 is more of a side issue, although not insignificant to a discussion of the expected collapse of nematicity with increasing doping.
In discussing the data at x=0.15 and x=0.30 (Supp. K), I would have liked to see the same methods of analysis used as in the discussion for x=0.08. For example, a plot of the first derivative of n(k) as shown for x=0.08 would have been very helpful. Instead, in Fig. 19b, the authors chose a method that they themselves had still classified in the main text of the manuscript as rather weak for identifying the Fermi surface contour. I personally find this picking of methods depending on the need and "hypothesis to be proven" somewhat problematic.

Now to the points of criticism of Referee #1 (I number them here as indicated in the Reply):
The Referee requested the additional data for x=0.15 and 0.30, which the authors also provided. However, the Referee is not convinced of the quality of the data and their analysis, nor of the Compton scattering method per se. However, in my eyes, the wording is not very serious and constructive in parts. In points 2 and 3, for example, he is surprised that on the one hand the data at x=0.08 (300K) and x=0.15 and on the other hand the data at x=0.08 (150K) and x=0.30 look amazingly similar. The corresponding n(k) maps are indeed very similar, but not identical. This similarity is the result of measurement and is not a reason to doubt the validity of the data! In particular, in the case of Compton scattering, although two n(k)-maps may look similar, this may in principle be due to different Fermi surfaces as well as additional effects. The authors have tried with some effort to give possible explanations for these experimental results, and in my view the interpretation of the x=0.30 data, as stated above, is possible but not quite convincing. I consider objection 4 of the Referee to be not very serious, reflecting at best an uneasy feeling.
The referee describes the data situation in point 5 as "sparse". Considering the high experimental effort (about 7-10 days measurement time per n(k)-map!) the offer of four such maps is more than

the vast majority of Compton papers offer. In point 6, the referee asks for even more data at lower temperatures and calls the authors' justification for not having measured them unconvincing. To my mind, this almost borders on spitefulness on the part of the referee, or he demands these additional data in ignorance of the effort involved. The authors rightly state that any more data would be beyond the scope of the original paper.
In addition, the referee claims that the authors used a "variety of parameters" to fit the data (point 5). This is obviously incorrect,, as the authors themselves point out. In fact, there are only two parameters (anisotropy and coupling strength along the c-axis). It is also shown that the coupling strength along the c-axis has no significant influence, effectively leaving only one parameter. For the remaining tight-binding parameters, one sticks to the established tight-binding model for cuprates and takes the previously published ARPES data as a basis, a procedure that, in my opinion, cannot be criticized.
Point 7: Here it is a case of statement against statement, which I cannot judge due to my lack of detailed knowledge of the cuprate system. However, I assume that the authors have studied the literature thoroughly and thus made a well-considered choice of their measurement temperatures.
Point 8: The referee criticizes an apparent lack of systematicity in the modelling of the data for x=0.15 and x=0.30, as well as different hopping parameters (one set for x=0.08, a second for 0.15 and 0.30. Although the authors justify their different choice of hopping parameters with their doping dependence (whether this is justified I cannot seriously judge, but it sounds plausible), it is also not obvious to me why one uses the same parameter set for x=0.15 and 0.30, but a different one for 0.08. Should one not use different parameters for all three cases? Apart from that, however, the logic of the modelling and the choice of parameters does not seem unreasonable or unsystematic to me. Therefore, I cannot understand the Referee's call for a more "transparent" approach. The authors' rationale for not using "doping-differential measurements" also seems conclusive to me: Since more than one aspect affecting the Fermi area and the occupation number density changes when the doping is increased, such differential spectra would not be very meaningful.

Conclusion:
Referee #1's comments, while at first glance seem understandable, are poorly reasoned and not constructive. The comments are also largely superficial, demonstrate a lack of thoroughness in the reading, and tend to follow a gut feeling of the Referee. Referee #2 was in favour of the manuscript from the beginning and recommends the revised form of the manuscript for publication after the first round of refereeing.
I find the paper mostly convincing despite the poor resolution of the measured data caused by the method, especially the main part related to x=0.08. I would only cut back on the interpretation of the data for x=0.30. Although the authors show here that they can explain the results within the framework of their data interpretation, a number of assumptions have to be made, which make the explanation sound less convincing. However, no contradiction arises with the core statement of the manuscript concerning the under doped regime at x=0.08, and I would therefore consider this point uncritical for publication, particularly since neither Referee '1 nor myself have a better explanation than the authors. Overall, these are new and extremely interesting results that are clearly presented and neatly discussed. The manuscript therefore absolutely deserves publication, despite the slight caveats for x=0.30 mentioned above, and Nature Communications is quite a suitable journal for this purpose. Since the publication has already been delayed for a long time due to the long referee process, I recommend publication of the manuscript as it stands without further changes!

Reviewer #4 (Remarks to the Author):

I am mainly concerned here with the manuscript in relation to the authors' response to reviewer 1's comments. I consider that the authors have sufficiently and thoroughly addressed or answered the comments.

I have read the paper, and find the conclusions to be justified. The demonstration of the effect on the observed Fermi surface caused by nematicity is convincing, and the explanation of the consequences of this for results from the Compton scattering measurements (as clearly depicted in figure 3) is sound. The Fermi surface as observed by Compton scattering is what I would expect in this scenario.

I agree that the doping dependence included in the supplementary material is consistent with expectation. I agree with the authors justification in terms of the Luttinger theorem.

I do not think that extra measurements or data are required to provide further support to the conclusions. Figure 4, and its comparison with figure 2, provides a convincing argument.

In conclusion, the paper presents a novel result that will be of interest to the community. The analysis and interpretation appears sound and the supplementary information underpins this. I recommend the manuscript for publication.
